# The Enzymatic Mechanism of OAS: How Metal Ions and Quantum Effects Help Activate Innate Immunity

**DOI:** 10.1021/acsomega.5c13236

**Published:** 2026-04-13

**Authors:** Pavel Kats, Xiaoyi Zhou, Jannik Wiebe, Ole Zeymer, Petra Baruch, Manuel H. Taft, Patrick Y. A. Reinke, Sebastian Günther, Alke Meents, Rune Hartmann, Dietmar J. Manstein, Roman Fedorov

**Affiliations:** † Institute for Biophysical Chemistry, Fritz–Hartmann–Centre for Medical Research, Hannover Medical School, Carl-Neuberg-Strasse 1, 30625 Hannover, Germany; ‡ Research Division for Structural Biochemistry, Hannover Medical School, Carl-Neuberg-Strasse 1, 30625 Hannover, Germany; § Center for Free-Electron Laser Science CFEL, 28332 Deutsches Elektronen-Synchrotron DESY, Notkestrasse 85, 22607 Hamburg, Germany; ∥ Department of Molecular Biology and Genetics, 1006 Aarhus University, Universitetsbyen 81, 8000 Aarhus, Denmark

## Abstract

2′-5′-Oligoadenylate synthetases (OAS) are crucial innate immune sensors that activate antiviral responses upon detecting viral double-stranded RNA. Understanding the molecular mechanism of OAS is vital for advancing immunomodulatory therapies. This study provides a detailed enzymatic mechanism of the OAS, integrating structural, kinetic, and quantum chemical analyses. Crystallographic data of the OAS1 postreactive complexes shed light on the geometry of OAS1 following product formation and dissociation, the sequential order of product release, and the pivotal role of divalent metal ions in catalysis. Our data reveal the unanticipated involvement of a third metal ion, which may play a transient supporting role in the catalytic cycle. Moreover, they highlight the central role of quantum mechanisms in the OAS function. Strikingly, substituting catalytic Mg^2+^ with Mn^2+^ ions increases the substrate binding rate 9-fold and activates OAS for catalysis. The results of this study are pertinent to the OAS/cGAS family of innate immune sensors and offer insights that can be applied to a broader class of nucleotidyltransferases, which play key roles in various biological processes.

## Graphical abstract

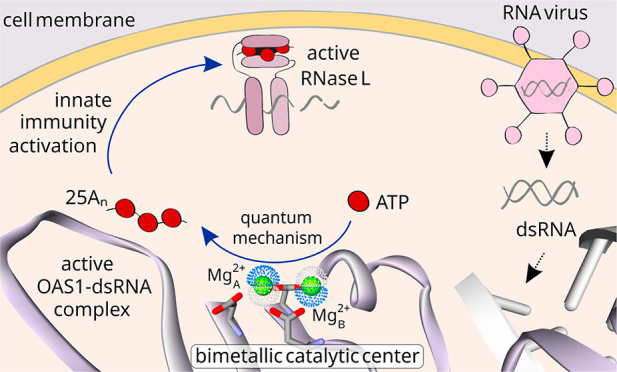

## Introduction

Innate immunity represents one of the most ancient, conserved, and powerful defense systems across the tree of life, predating the adaptive immune responses unique to vertebrates.^1,2^ The core components of human innate immunity originated around one billion years ago^3,4^ and can be traced to primitive organisms such as bacteria and sea sponges.^5,6^ These organisms developed innate immune sensors capable of detecting and responding to microbial pathogen-associated molecular patterns (PAMPs), a trait that has been inherited by higher organisms, including humans.^1,7–10^ A key factor in the evolutionary success of these mechanisms is the cross-species conservation of pattern recognition receptors (PRRs), which underscores their efficacy in protecting against pathogens across taxa, from invertebrates to mammals.

One class of PRRs is given by cytosolic sensors, which recognize intracellular pathogens, particularly viruses.^11^ These sensors play a pivotal role in activating the downstream immune responses. Among them, 2′-5′-Oligoadenylate synthetase (OAS) and cyclic GMP-AMP (cGAMP) synthase (cGAS) constitute a family of cytosolic PRRs.^12–14^ Both enzymes are activated by recognizing nucleic acids and play essential roles in the antimicrobial response. Upon activation by double-stranded (ds) DNA, cGAS synthesizes cGAMP second messengers that activate the Stimulator of Interferon Genes (STING) pathway, resulting in the production of type I interferons and other pro-inflammatory cytokines.^15^ Similarly, OAS is activated by binding to dsRNA (Figure 1) and synthesizes 2′-5′-oligoadenylates (25A_*n*_) in a stepwise manner, sequentially adding AMP moieties (Figure S1).^12,14,16^ These 25A_*n*_ molecules activate RNase L, an endoribonuclease that degrades viral and host RNA, thereby limiting viral replication.^17^ Together, the RNase L and STING pathways form a critical first line of defense against viral infections, highlighting the essential role of nucleic acid sensing in innate immunity. In human, the OAS protein subfamily consists of the three catalytically active enzymes OAS1, OAS2, and OAS3, and the OASL protein lacking enzymatic activity.^12,18^ The modulation of the OAS as a sensor of innate immunity holds significant therapeutic potential. OAS is implicated in various clinical conditions where the immune system is either compromised or overactive. Notably, OAS plays a critical role in viral infections.^19^ For instance, OAS1 was shown to be a potent inhibitor of SARS-CoV-2 replication,^20,21^ and treatment with chemically synthesized 25A_*n*_s has been proposed to prevent severe acute respiratory syndrome.^22^ Beyond its antiviral functions, OAS has been implicated in the regulation of autoimmune disorders^23–25^ and is associated with interferonopathies,^26^ malaria,^27^ heart failure,^28^ elderly stroke,^29^ and various cancers.^30–32^ Modulating the OAS activity can boost the host’s antiviral defenses, as in COVID-19, or mitigate inappropriate immune responses in autoinflammatory diseases and cancer. However, the development of effective immunomodulatory agents targeting the OAS requires a detailed understanding of its complex enzymatic mechanisms.

**Figure 1:**
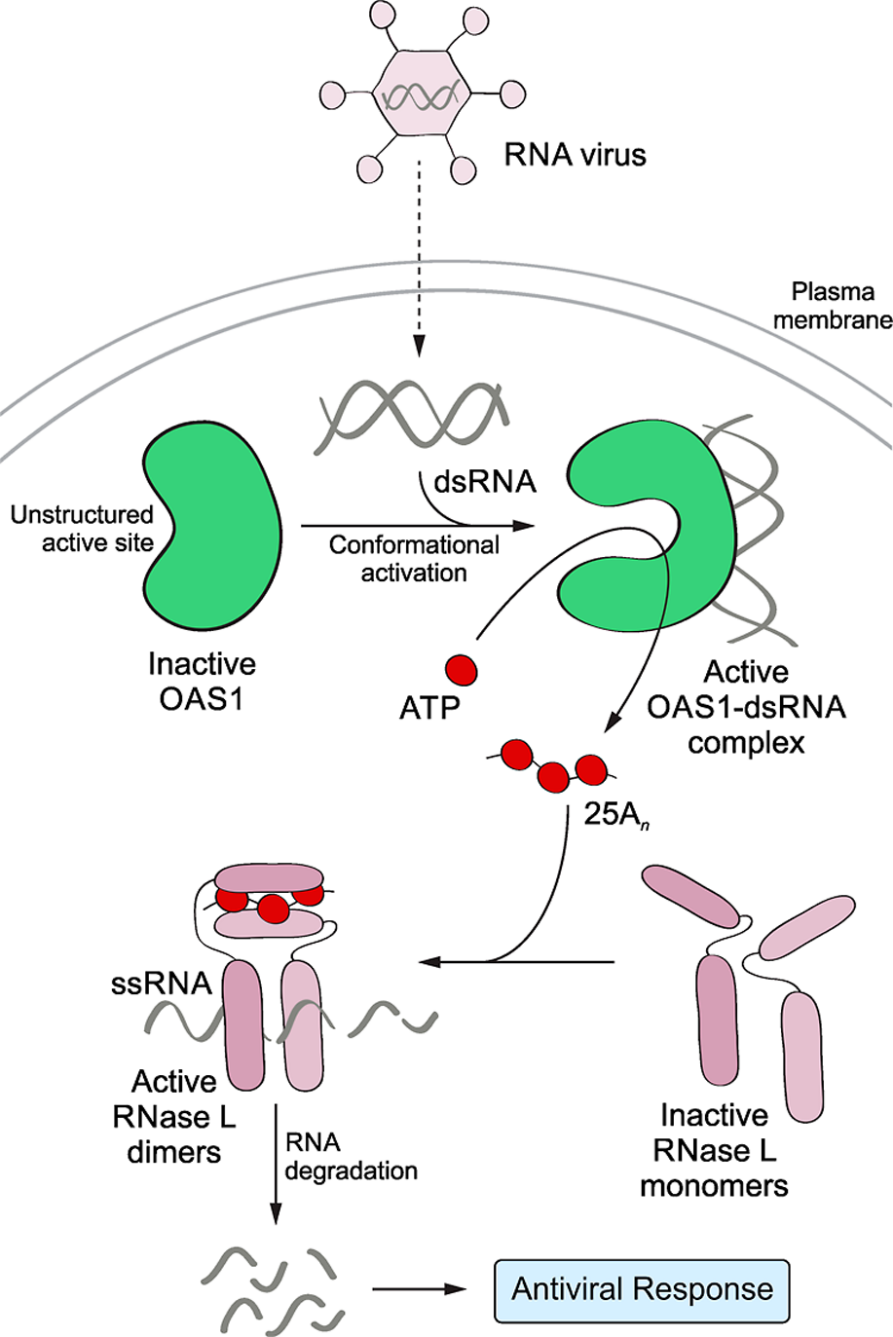
Schematic overview of 2′-5′-oligoadenylate synthetase (OAS)-RNase L signaling. Upon infection, the pathogen’s genetic material is released into the cytosol, where it can be recognized by the innate immune sensor OAS1. The interaction with the dsRNA activates the enzymatic function of OAS1. The activated OAS1–dsRNA complex binds ATP substrates to generate 2′-5′-linked oligoadenylates (25A_*n*_), which act as second messengers. The 25A_*n*_ molecules with *n* ≥ 3 facilitate the formation of the RNase L dimers possessing ribonuclease activity. The RNaseL dimers degrade viral and intracellular RNA to initiate an antiviral response.

Previously, we investigated the molecular mechanism underlying nucleic acid-induced activation of porcine OAS1 (pOAS1), including the distinct roles of nucleic acids and substrates, the sources of 2′-specificity of product formation, and the basis of functional divergence between OAS1 and cGAS.^14^ Structural insights into the enzymatic cycle of OAS1 encompass multiple states: the apo-state (PDB: 4RWQ), the RNA-bound state (PDB: 4RWP), the RNA- and AMP-donor-bound state (PDB: 4RWO), and the fully formed prereactive state (PDB: 4RWN), which includes both substrates and metal ions in the catalytic center of the OAS–dsRNA complex (Figure S2). The sequential mechanism of pOAS1 activation based on crystallographic, kinetic, and computational studies^14^ suggests that the RNA-binding causes a major restructuring of the N-terminal lobe of the enzyme, which results in a favorable conformation for the binding of substrates and magnesium cofactors, and subsequent catalysis. A recent comprehensive kinetic study of the human OAS1 (hOAS1) reaction^33^ has shown that the dynamic properties of OAS1 allow alternative pathways leading to the formation of a catalytically active complex. As porcine and human OAS1 share 75% sequence similarity and identical active sites, their mechanisms are analogous, and their studies complement each other.

The events that follow the formation of the prereactive state of the OAS have not yet been studied at the molecular level. There is a lack of structural and mechanistic data for product formation and release. These processes are critical for OAS function, as they determine the intracellular concentration of 25A_*n*_s, which in turn regulates the activation of RNase L.^19^

To address the gaps in structural knowledge of OAS product formation and gain new insights into the catalytic mechanism of the OAS1 reaction, we determined crystal structures of two postreactive states of pOAS1 in complex with dsRNA and products 25A_2_ and PP_i_ (Figure S2). The reported structures offer the first insights into the mechanisms of product formation and release in OAS1 and suggest a different order of product dissociation compared to other nucleotidyltransferases (NTases).^34,35^ To help interpret experimental structural data and elucidate their link with the electronic effects of the OAS reaction, we conducted a quantum chemical study of the OAS reaction pathway. These combined experimental and computational studies offer important insights into the mechanism of the OAS reaction and the role of the catalytic center in the biosynthesis of innate immune second messengers. Specifically, our findings highlight the critical role of metal ions in all stages of the OAS reaction, from catalysis to substrate binding and product release. We also demonstrate how quantum-mechanical electronic effects ensure the functional activity of the OAS.

To further explore the impact of metal properties on the OAS reaction, we performed crystallographic, kinetic, and quantum chemical studies of pOAS1 in the presence of manganese ions. These studies revealed the role of the outer electron shells of manganese ions in enhancing the affinity, binding rate, stability, and activity of the substrates at the catalytic center. Moreover, we discovered a third transient metal ion involved in the catalytic effects of the OAS. Given the high similarity between the active sites of the OAS and cGAS, as well as the catalytic domains of other NTases, these results are relevant to a wide range of essential enzymes involved in DNA replication, repair, transcription, RNA processing, viral replication, and signal transduction.

## Results

### Crystal Structures of OAS1 Postreactive States

#### pOAS1·dsRNA·25A_2_·PP_i_·Mg_**A**_^2+^·Mn_**B**_^2+^ Complex Structure

The postreactive complex was derived from crystals of the prereactive pOAS1 complex (PDB: 4RWN)^14^ by replacing the nonreactive ATP-analog adenosine-5′-[(α,β)-methyleno]-triphosphate (ApCpp) with ATP. This substitution initiated the catalytic reaction within the crystals. The reaction products in the active site were stabilized by the inclusion of manganese ions. The crystals diffracted to a resolution of 1.8 Å, and the structure was solved by molecular replacement using the protein and dsRNA components of the prereactive complex (PDB ID: 4RWN) as the starting model. The refined structure yielded a high-quality electron density (Table S1) with excellent resolution for all components of the complex. Notably, strong electron density was observed for both the 25A_2_ and PP_i_ products within the active site of the OAS (Figure 2). The overall conformation of pOAS1 in the postreactive complex (Figure 2A) closely resembles that of the prereactive state, with an r.m.s.d. of just 0.5 Å for C_α_ atoms. Superposition of the complete protein-dsRNA complexes in both states revealed no significant changes in the nucleic acid binding mode. The primary structural differences in the protein are found in the flexible loops β3–β4 and α6–α7, with maximum main chain shifts of 4.7 Å and 1.0 Å, respectively (Figure S3A). These shifts, along with minor conformational changes throughout the rest of the protein, result in a slightly more closed conformation of pOAS1 in the postreactive state. This conformational change leads to a reduction in both the volume and inner surface area of the active site cavity, from 1757 Å^3^ and 1205 Å^2^ in the prereactive state to 1489 Å^3^ and 1094 Å^2^, respectively.

**Figure 2:**
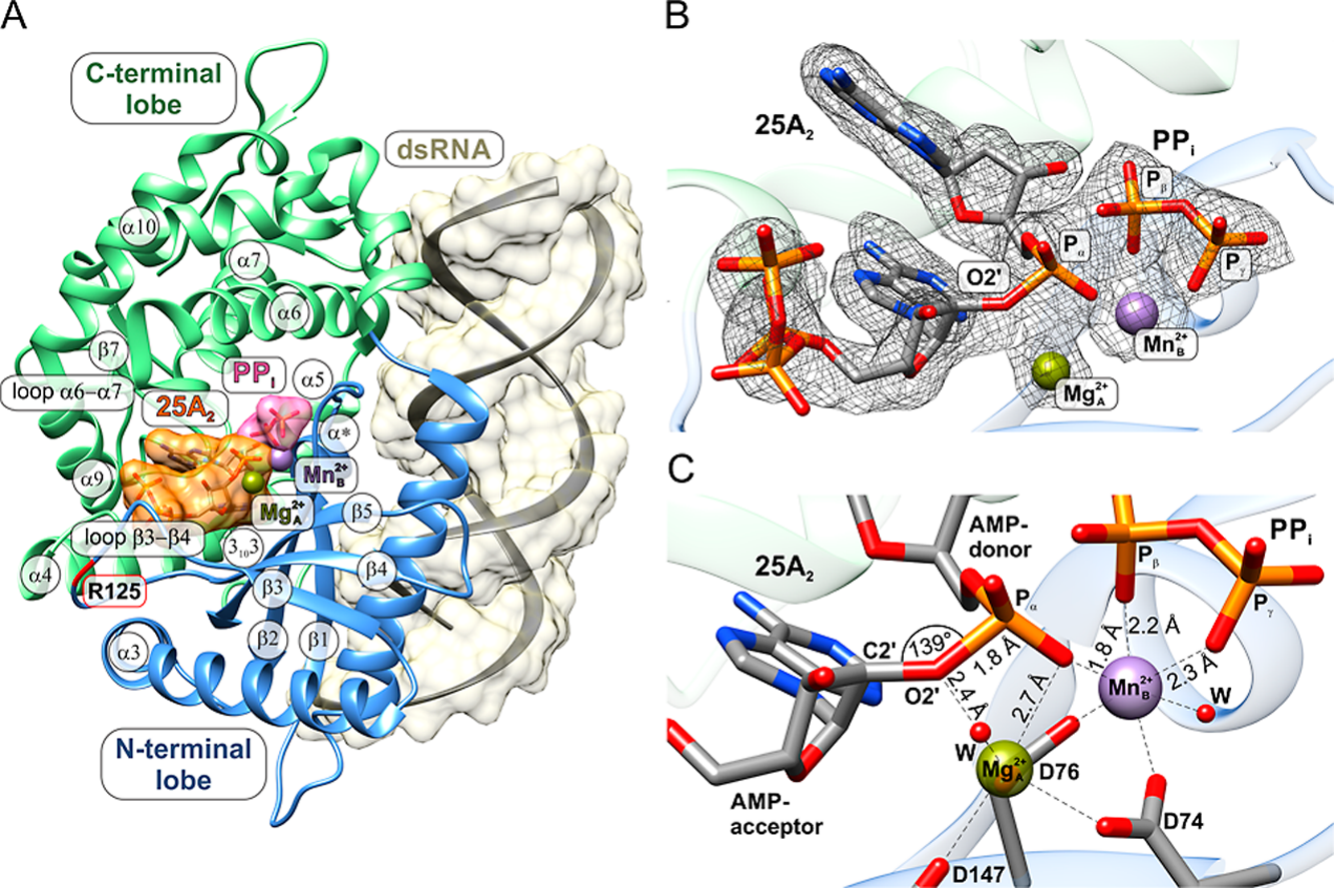
Postreactive pOAS1·dsRNA·25A_2_·PP_i_·Mg_A_^2+^·Mn_B_^2+^ complex with active site-bound OAS products 25A_2_ and PP_i_. (A) Overview of the postreactive pOAS1 complex, encompassing the protein, the dsRNA, and products 25A_2_ and PP_i_, along with the Mg_A_^2+^ and Mn_B_^2+^ ions bound to the catalytic center. Key secondary structure elements of OAS1 are labeled, with residue R125 enclosed in a red box. The dsRNA and the products 25A_2_ and PP_i_ are shown with a semitransparent surface. (B) Experimental 1.8 Å 2*F*_o_–*F*_c_ omit electron density at the 1.0 σ level for the catalytic center of pOAS1, showing the binding mode of 25A_2_ and PP_i_ in coordination with one magnesium ion Mg_A_^2+^ and one manganese ion Mn_B_^2+^. (C) Close-up representation of the metal-coordinated catalytic center of pOAS1 in the postreactive complex with the catalytic triad residues D74, D76, and D147, showing the geometric properties of the products 25A_2_ and PP_i_. Red spheres labeled with “W” represent water molecules.

The active site of pOAS1 forms a deep, elongated cavity situated at the interface between the N- and C-terminal lobes (Figures 2A and S4). This cavity is shaped by several secondary structure elements, including α*, β2, α3, β3–β4 (including the loop), β5, loop α4-3_10_3, 3_10_3, α5, α6–α7 (including the loop), loops α9–β7 and β7–α10, and α10. The β3–β4 and α6–α7 loops gate the outermost part of the active site. A total of 69 residues in pOAS1 contribute to the formation of the active site, with roughly equal contributions from polar (36%), charged (34%), and hydrophobic (30%) residues. The catalytic reaction center of the OAS is located within a narrow well at the deepest part of the active site cavity. The core of the catalytic center, which coordinates the reacting atoms, consists of the catalytic triad D74 (β2), D76 (β2), D147 (β5), along with two metal ions (Figure 2B,C). The catalytic triad and metal cofactors are surrounded by strictly conserved residues from the β1–α*–β2, β4–β5, α5–α7, and β7–α10 loop regions, which play a role in coordinating the OAS reaction products.

The positioning of the 25A_2_ and PP_i_ products in the active site of pOAS1 is very similar to the ApCpp substrates in the prereactive state (Figure S3B), consistent with the principle of least motion in chemical reactions.^36^

The electron density for 25A_2_ and PP_i_ products at the catalytic center is exceptionally well-structured and strong (Figure 2B). Nearly all atoms of the products are well resolved and unambiguously positioned at a 1.0 σ density level, except for a slightly disordered P_β_ phosphate from the AMP acceptor. The bond between the O2′ atom of the AMP acceptor and P_α_ of the AMP donor is clearly visible. A peak of 6.5 σ in the 2*F*_o_–*F*_c_ omit electron density for 25A_2_ is located on the P_α_ group from the AMP donor. For the PP_i_ byproduct, the 2*F*_o_ *-*–*F*_c_ omit electron density maxima of 8.8 σ are located on the P_β_ and P_γ_ atoms, which is 35% higher than the peak for 25A_2_. This suggests that, in the postreactive state, the main product has a weaker binding affinity for the OAS compared to the byproduct. The same conclusion follows from the comparison of temperature *B*-factors, which are above average for P_α_ and below average for P_β_–Pγ groups. In contrast, in the prereactive state, all AMP donor phosphates exhibit comparable electron density peaks and comparable below-average *B*-factors.^14^ The metal ions coordinating the products at the catalytic center also display strong electron density peaks. In this structure, the electron density analysis confirms that the Mg_B_^2+^ ion has been replaced by a manganese ion Mn_B_^2+^, which shows a higher electron density peak than the Mg^2+^ ion at site A, consistent with the number of electrons’ ratio in Mn^2+^ and Mg^2+^ ions. Additionally, the replacement of the Mg_B_^2+^ ion by Mn^2+^ is confirmed by the anomalous density analysis, which revealed an anomalous peak at site B, while no anomalous signal was detected at site A (Figure S5A). The electron density also allows for unambiguous positioning of the catalytic triad residues D74, D76, and D147, which coordinate the metal ions. The metal ions coordinate two water molecules, whose electron densities are comparable to those of protein oxygens and ligands within the metal coordination spheres.

The conformation of the 25A_2_ product bound to the catalytic center closely resembles that of the substrates in the prereactive state (Figures 2C and S3B). In the postreactive state, the nucleosides of the AMP-donor and AMP-acceptor are positioned approximately 90° relative to each other. The nucleoside of the AMP donor interacts with residues K65 (α*), S186 (helix 3_10_3), Q193 (α5), P228 (loop α6–α7), D300, and G306–V308 (loop β7–α10). Meanwhile, the nucleoside of the AMP acceptor is coordinated by residues V78 (β2), A130 (loop β3–β4), L149 (β5), S186–T187 (helix 3_10_3), T190, and Q193 (α5), along with the Mg_A_^2+^ ion (Figure S6).

The triphosphate moiety of the AMP acceptor is extended into a wider part of the active site cavity, where it remains fully solvent-accessible. It forms three H-bonds with residues R125 and R129 from loop β3–β4 (Figures 2A and S3A). As demonstrated in our previous study,^14^ mutation of R129 to alanine reduces enzymatic activity to 62% ± 4% of wild-type levels, highlighting its role in AMP acceptor stabilization. Notably, the interaction with R125 is absent in the prereactive state and becomes possible due to the more closed conformation of the active site, including the loop β3–β4 in the postreactive complex. To assess the role of R125 in product stabilization, we generated an R125A mutant. This substitution resulted in a significant reduction in enzymatic activity to 78.7% ± 5.2% of wild-type pOAS1 levels (Figure S7).

The main structural differences between the substrates and the product in the catalytic center are located in the vicinity of the reacting atoms, O2′ of the AMP acceptor, and P_α_ of the AMP donor. The formation of the covalent bond O2′–P_α_ (Figure 2B) is accompanied by a shift of the AMP acceptor ribose toward the AMP donor, Walden inversion of the P_α_ group, and an increase in the C2′–O2′–P_α_ angle from 113° to 139° (Figure 2C). The elongated shape of the electron density along the P_α_–O1A bond indicates the likely presence of a proton on the oxygen of the α-phosphate. Following the reaction, the O2′ and P_α_ groups remain within the coordination spheres of the metal cofactors, forming two coordination bonds with Mg_A_^2+^ and one with Mn_B_^2+^. The strongest interaction (1.8 Å) between 25A_2_ and the catalytic center is a coordination bond with Mn_B_^2+^. In contrast, the coordination bonds of 25A_2_ with Mg_A_^2+^ have longer distances and a distorted angle between the O2′–Mg_A_^2+^ bond and the equatorial plane of the Mg_A_^2+^ coordination sphere. These distortions result from steric effects induced by the formation of the O2′–P_α_ covalent bond. The distorted coordination interactions between biadenylate and Mg_A_^2+^ indicate that the binding of the AMP acceptor part of 25A_2_ to the catalytic center is significantly weaker than in the prereactive state. The latter is confirmed by the experimental electron density analysis described above.

The decrease of the product binding affinity following the O2′–P_α_ bond is likely to facilitate product dissociation. Given the tightly confined topology of the 25A_2_ binding site, the product can only move in one direction, toward the larger volume of the active site, approaching the gating loops β3–β4 and α6–α7. The steric constraints of the active site preclude the direct relocation of 25A_2_ to the acceptor site for the subsequent rounds of the OAS-catalyzed step-growth oligomerization. Thus, the crystal structure of the OAS postreactive state suggests that after product formation, 25A_2_ dissociates from the catalytic center into a larger active site volume, where it can reorient and rebind to the acceptor site for the next oligomerization step.

The binding mode of PP_i_ at the active site of the protein in the postreactive complex is hardly changed compared to the P_β_–P_γ_ moiety of the AMP donor substrate in the prereactive state (Figure S3B). The byproduct is stabilized by direct contacts with the residues G61–S62 and S73 (α*), K212 (α6), Q229 (α7), one ethylene glycol molecule, and the Mn_B_^2+^ ion (Figure S8). The contact of PP_i_ with K212 becomes closer in the postreactive complex, indicating enhanced interaction and possible proton transfer between them. Additionally, PP_i_ engages in a solvent-mediated hydrogen-bond network involving residues T67 (α*), Q216 (α6), K219 (α6), and E233 (α7) (Figure S8). Both the P_β_ and P_γ_ groups remain in the coordination sphere of the Mn_B_^2+^ ion. While these coordination bonds are slightly elongated compared to the prereactive state, they still maintain a nearly perfect octahedral geometry of the Mn_B_^2+^ coordination sphere. The highly stabilized conformation of the PP_i_ in the postreactive state complex suggests that following the catalytic reaction, the byproduct binds to the catalytic center significantly stronger than 25A_2_. This observation is further supported by the experimental electron density analysis described above.

#### pOAS1·dsRNA·25**A**_2_^diss^·PP_i_·Mg_**A**/**B**_^2+^ Complex Structure

In an attempt to obtain a native Mg_A/B_^2+^-bound postreactive complex structure of the OAS, we performed a series of crystallographic experiments. These included cocrystallization of pOAS1·dsRNA complex with ATP or its modified analogs, as well as direct or EDTA-mediated substitution of ApCpp with ATP in the prereactive pOAS1 complex crystals. The resulting crystals produced high-quality X-ray diffraction data and electron density maps with high-resolution limits from 2.1 to 2.7 Å. However, none of these structures revealed the presence of the 25A_2_ product within or near the Mg^2+^-bound catalytic center, although the PP_i_ byproduct was consistently observed. The only Mg_A/B_^2+^-bound postreactive complex containing both reaction products was obtained by crystallizing the pOAS1 in the presence of dsRNA and the fluorescent ATP analog 2′-/3′-*O*-(*N*-methylanthraniloyl)-ATP (mant-ATP) followed by flash-freezing the crystals in liquid nitrogen. This structure, solved by molecular replacement using the prereactive complex (PDB: 4RWN) as the starting model, was refined to a 2.7 Å resolution (Table S1). The electron density map revealed that the PP_i_ byproduct remained bound at the catalytic center, while the main product, 25A_2_, had dissociated toward the outer part of the OAS active site, forming a weak contact with the gating loops β3–β4 and α6–α7 (Figure 3).

**Figure 3:**
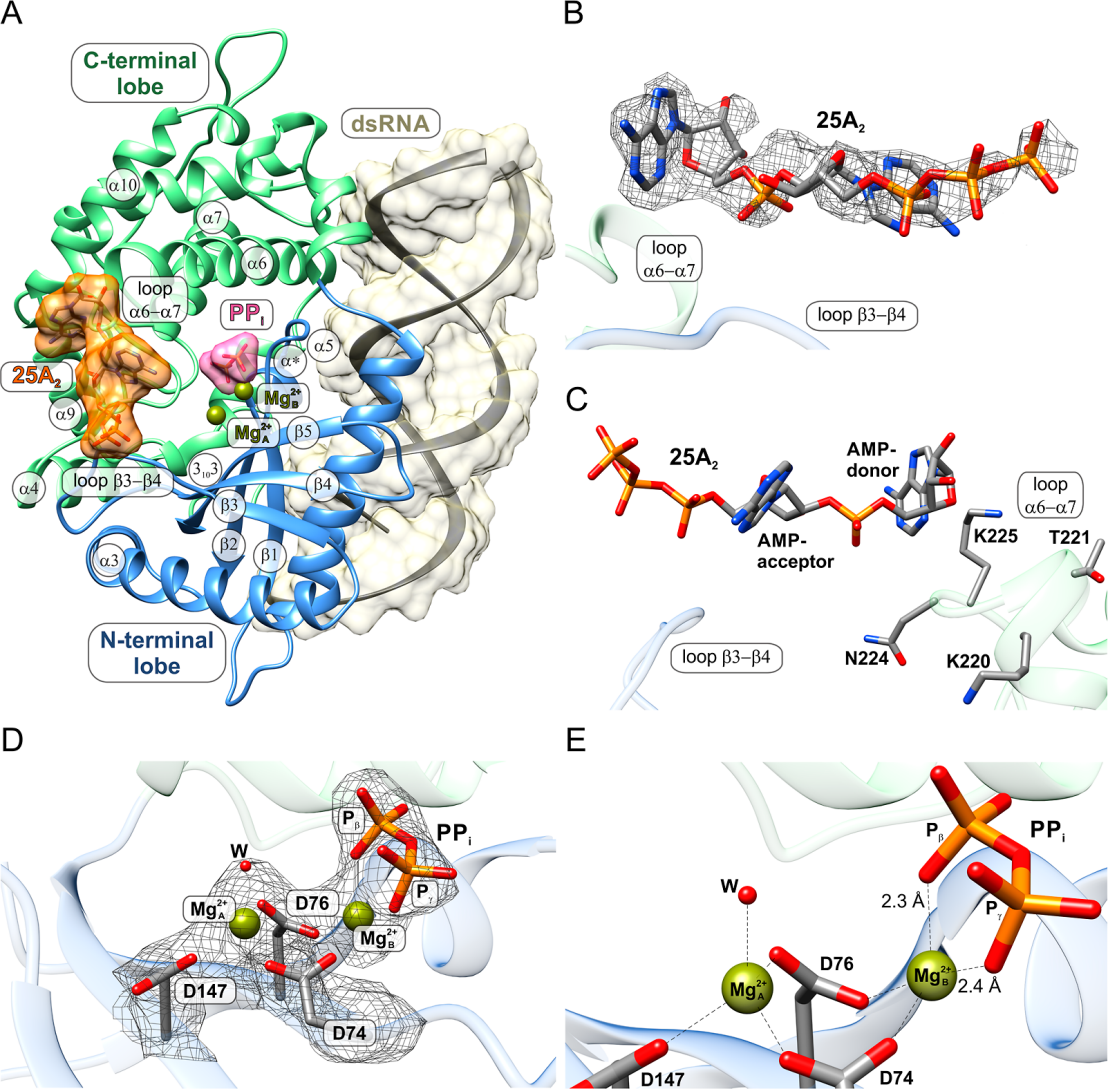
Postreactive pOAS1·dsRNA·25A_2_^diss^·PP_i_·Mg_A/B_^2+^ complex with dissociated 25A_2_ and active site-bound PP_i_. (A) Overview of the 25A_2_-dissociated postreactive pOAS1 complex, including the protein, the dsRNA, and both products 25A_2_ and PP_i_, together with two magnesium ions Mg_A/B_^2+^. Key secondary structure elements of pOAS1 are labeled nearby. The dsRNA and the products 25A_2_ and PP_i_ are shown with a semitransparent surface. (B) Experimental omit electron density map after density modification procedure at the 1.0 σ level for the dissociated 25A_2_ biadenylate. (C) The binding mode of 25A_2_ at the area between OAS loops β3–β4 and α6–α7. (D) Experimental 2*F*_o_–*F*_c_ omit electron density at the 1.0 σ level for the catalytic center of OAS in the 25A_2_-dissociated state, showing the binding mode of PP_i_ coordinated with the magnesium ions Mg_A/B_^2+^. (E) Close-up representation of the metal-coordinated catalytic center of pOAS1 in the 25A_2_-dissociated postreactive complex with the catalytic triad residues D74, D76, and D147, showing the geometry of the PP_i_ binding. Upon dissociation of 25A_2_, both magnesium ions change their coordination number from six to four. Red spheres labeled with “W” in (D,E) represent water molecules.

At the protein tertiary structure level, this 25A_2_-dissociated complex closely resembles the previously described postreactive state complex with catalytic center-bound 25A_2_ (Figures 2A and 3A). There are no major changes either for protein chains (0.6 Å r.m.s.d. for C_α_ atoms) or for the binding mode of dsRNA. However, compared to the prereactive and 25A_2_-bound postreactive states, the 25A_2_-dissociated complex adopts a more open conformation (Figure S9A), similar to the pOAS1·dsRNA complex (PDB: 4RWP). The most pronounced structural change in the N-terminal lobe is observed for the main chain of the central β-sheet, which moves up to 1.0 Å away from the nucleic acid and shifts the entire catalytic center while preserving its internal geometry. The C-terminal lobe undergoes less significant rearrangements (≤0.5 Å), but these collectively contribute to the active site expansion (Figure S9B,C). As a result, the active site volume increases by 25% compared to the 25A_2_-bound postreactive complex and by 6% relative to the prereactive state, potentially facilitating product dissociation.

As mentioned above, no electron density for 25A_2_ was detected at the catalytic center (Figure 3D). Instead, weak and partly disordered electron density for the main product was identified at the outermost region of the active site, between loops α6–α7 and β3–β4 (Figure 3B), suggesting a low-affinity, transient binding state. The *2F*_o_–*F*_c_ omit electron density peak of 1.1 σ is centered on the adenyl moiety of the AMP donor, while the mant-groups were absent, likely as a result of radiation-induced cleavage. The 25A_2_ product adopts a stretched conformation, with the AMP donor positioned near the loop α6–α7 and the AMP acceptor near the loop β3–β4 (Figure 3C). Binding interactions include contacts with residues K220, T221, G223 (α6), N224, and K225 (α6–α7), along with a single hydrogen bond to the Q139 side chain from a neighboring unit cell. Most of these contacts are weak hydrophobic or van der Waals interactions, insufficient to firmly stabilize 25A_2_ in this region. Nevertheless, the fact that a weakly bound intermediate product is observed near the active site gating loops suggests the presence of a steric barrier that may influence product release. This barrier might help redirect 25A_2_ toward the catalytic center for the subsequent rounds of the OAS-catalyzed oligomerization.

Unlike the main product, the electron density for the byproduct is located at the same site of the catalytic center as the PP_i_ moiety in the previous postreactive state structure (Figure 3D). This density is well-defined, allowing for unambiguous positioning of PP_i_ at the 1.0 σ level. The average *B*-factor for the PP_i_ moiety is 45% higher than that of the whole structure (Table S1), in contrast with the postreactive and prereactive state complexes, where the byproduct is better stabilized and has below-average *B*-factors. This suggests a decreased PP_i_ binding affinity following dissociation of the main product. The binding mode of PP_i_ closely resembles its binding in the 25A_2_-bound postreactive state (Figures 2C and 3E). The PP_i_ is stabilized through direct interactions with residues G61, S62, K65, S73 (α*), K212 (α6), Q229 (α7), E233 (α7), and two coordination bonds with Mg_B_^2+^. Although both the P_β_ and P_γ_ groups remain coordinated to the Mg_B_^2+^ ion, their coordination bonds are more elongated compared to the prereactive and 25A_2_-bound postreactive complexes.

The electron density also enables unambiguous positioning of the catalytic triad residues D74, D76, and D147, as well as coordinated magnesium ions Mg_A_^2+^ and Mg_B_^2+^. Notably, the electron density peaks for these ions are significantly smaller than those observed in the 25A_2_-bound postreactive and prereactive OAS complexes, indicating that metal binding to the active site is weakened after 25A_2_ dissociation. This effect is accompanied by a transition of Mg_A_^2+^ and Mg_B_^2+^ coordination geometries from octahedral to tetrahedral.

The observed partial destabilization of the catalytic center associated with a breaking of magnesium coordination symmetry is consistent with previous observations,^14^ where an increase in coordination symmetry from tetrahedral to octahedral led to an increase in the stability of the catalytic center during the formation of the prereactive state. The distortion of tetrahedral coordination symmetry observed for both magnesium ions in our structure further supports the conclusion that the binding affinity has weakened. These effects likely contribute to the dissociation of the byproduct and metal ions from the catalytic center, enabling it to reset for the next enzymatic reaction cycle.

### Quantum Chemical Studies of OAS1 Reaction and Postreactive States

To better understand the formation of postreactive states and the factors driving product release in the OAS, we performed an experiment-guided quantum chemical (QC) analysis of the geometries and electronic properties of the OAS catalytic center along the reaction pathway using ab initio Density Functional Theory (DFT). Details of the QC analysis of the OAS1 reaction are provided in the Methods section and the Supporting Information.

The QC studies reveal that the catalytic center in the prereactive state possesses optimal geometry, charge distribution, and electronic properties for initiating the S_N_2 reaction (Figure 4A,B). The QC analysis shows that the nucleophile atom O2′ is already deprotonated in the prereactive complex. This conclusion is supported by high-resolution X-ray crystallographic structures (PDB-ID: 4RWN, 9NYB, and unpublished data), which consistently reveal the O2′–Mg_A_^2+^ distances and coordination geometries compatible only with the deprotonated form of the substrate. Moreover, when O2′ is artificially protonated, and the resulting system is optimized using ab initio QC methods, the AMP acceptor adopts an altered binding geometry, in apparent disagreement with the experimental structures.

**Figure 4:**
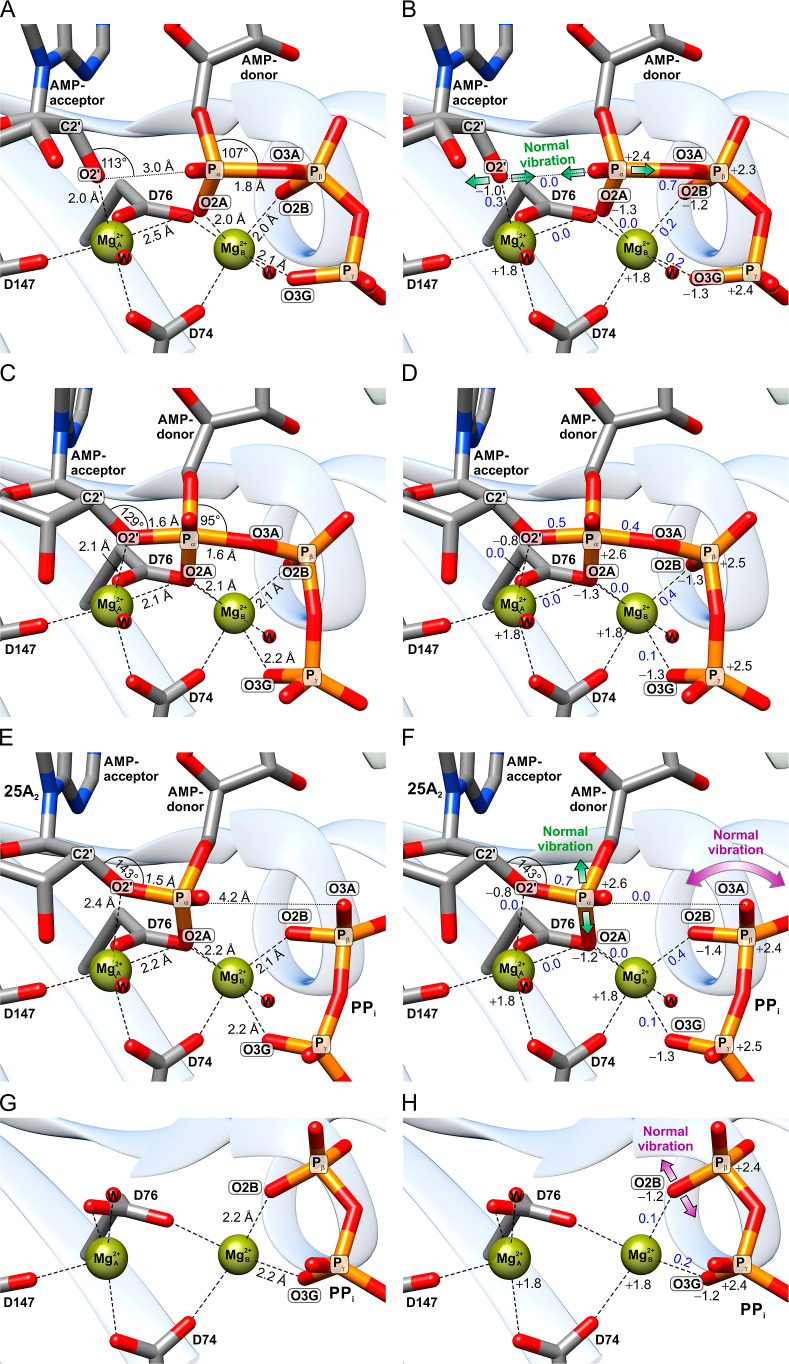
QC-geometry (left column) and electronic properties (right column) of the pOAS1 catalytic center in the prereactive (A,B), transition (C,D), postreactive (E,F), and PP_i_ (G,H) states. The carbon, nitrogen, oxygen, phosphorus, and magnesium atoms are shown in gray, blue, red, orange, and green colors, respectively. Hydrogen atoms are omitted for clarity. The DFT natural bond orbital (NBO) charges and bond orders in the right column are shown in black and blue, respectively. The green arrows in panel (B) indicate the QM normal mode, which describes the reaction pathway of OAS. The purple arrow in panel (F) indicates the QM normal mode describing the separation of the P_β_–P_γ_ group from 25A_2_ upon the product formation via the rotational movement of PP_i_. The green arrow in panel (F) indicates the QM normal mode, corresponding to the release of the main product from the catalytic center into the main active site volume. The purple arrow in panel (H) shows the QM normal mode corresponding to the exit of the β-phosphate from the coordination sphere of Mg_B_^2+^.

The positions of the reacting groups are stabilized by coordination bonds with the magnesium cofactors. These magnesium ions contribute to OAS catalysis through several mechanisms: they ensure the proximity and orientation of the reagents, induce bond strain on the P_α_ group of the AMP donor, and electrostatically activate the reacting groups. The coordination bonds between the cofactors and the reagents involve both electrostatic interactions and electron exchange (Figure 4B).

The electron exchange is facilitated by the overlap between the molecular orbitals of the interacting groups, which is reflected in the positive bond order values. The P_α_ group of the AMP donor exhibits zero bond orders with magnesium ions, indicating a higher lability of the α-phosphate compared to the P_β_ and P_γ_. This property is confirmed by the experimental electron density analysis of the prereactive state crystal structure (PDB-ID: 4RWN). The greater lability of α-phosphate likely facilitates its movement toward the nucleophile during the reaction.

The lack of chemical bonding between the α-phosphate and Mg_B_^2+^ can be attributed to the deviation of the O2A atom from the axial position within the octahedral coordination sphere of the metal ion, resulting in a loss of molecular orbital overlap. Furthermore, the QM normal mode vibrational analysis (QM-NMA) indicates that nucleophilic attack in the OAS S_N_2 reaction occurs along a thermally activated, low-frequency normal vibration, facilitating the reaction (Figure 4B).

The transition state (TS) of the OAS reaction exhibits a trigonal-bipyramidal geometry, characteristic for the S_N_2 reaction mechanism (Figure 4C). The formation of the chemical bond between the O2′ of the AMP acceptor and the P_α_ of the AMP donor drives the movement of these atoms toward each other, away from their prereactive positions. This movement disrupts the octahedral symmetry of the Mg_A_^2+^ coordination sphere, weakening its chemical bonding and electrostatic interaction with O2′ (Figure 4D). Simultaneously, the O2′–P_α_ interaction becomes partially covalent while the bond between P_α_ and the leaving group weakens. During the TS formation, a proton is transferred to the O1A oxygen of the AMP donor P_α_ group from the nearest water molecule in the coordination sphere of Mg_A_^2+^. This proton transfer lowers the energy and increases the stability of the TS. The calculated free energy barrier for the pOAS1 catalytic reaction (Supplementary Results) aligns well with the experimental kinetic data for pOAS1,^14^ hOAS1,^33^ and with similar kinetic data and QC calculations from related reactions within the same superfamily.^34,37,38^ These findings validate the accuracy of our QC model for the pOAS1 catalytic reaction.

The formation of the postreactive state from the TS proceeds through a further approach of the O2′ and P_α_ atoms, followed by the stereochemical Walden inversion of the α-phosphate, the breaking of the P_α_–O3A bond, and the separation of the pyrophosphate group from the newly formed 25A_2_ product (Figure 4E). The O1A oxygen of the reactive P_α_ group remains protonated in 25A_2_. The product formation leads to structural and electronic changes in the catalytic center, resulting in the destabilization of the 25A_2_ position (Figure 4E,F). The weakly bound 25A_2_ can dissociate from the catalytic center into the main volume of the active site cavity, as predicted by QM-NMA (Figure 4F) and molecular quantum dynamics (MQD) calculations. Brownian motion of solvent-exposed regions of 25A_2_ is likely to facilitate the dissociation process. It is noteworthy that the product dissociation vector resulting from QM analysis aligns with the experimentally observed dissociation channel in the protein structure, suggesting a synergy between the quantum properties and protein architecture in the product release mechanism.

The PP_i_ separates from 25A_2_ via a rotational movement, preserving the coordination and interactions of the pyrophosphate oxygens O2B and O3G (Figure 4E,F). Proton transfer from K212 to PP_i_ occurring during this process helps stabilize the resulting PP_i_ orientation and explains the enhanced PP_i_–K212 interaction observed in the experimental complex structure.

The higher stabilization of pyrophosphate in the postreactive state, compared to 25A_2_, explains why 25A_2_ is released first from the catalytic center, as observed in the crystal structure of the pOAS1·dsRNA·25A_2_^diss^·PP_i_·Mg_A/B_^2+^ complex. The QC analysis of the postreactive state properties fully aligns with experimental electron density and stereochemical analyses of pOAS1·dsRNA·25A_2_·PP_i_·Mg_A_^2+^·Mn_B_^2+^ and pOAS1·dsRNA·25A_2_^diss^·PP_i_·Mg_A/B_^2+^ crystal structures. The release of 25A_2_ leads to a decrease in bond orders and electrostatic interactions between the pyrophosphate and the Mg_B_^2+^ ion, and the emergence of a normal mode corresponding to the PP_i_ dissociation (Figure 4G,H). These effects contribute to weakening of the PP_i_ interaction with the catalytic center and complement the results of experimental crystallographic analysis (Figure 3).

### The Effect of Manganese on the OAS Reaction

As demonstrated in previous studies, OAS can utilize manganese for its enzymatic reaction.^39^ More recently, it has been identified that manganese can substitute catalytic magnesium in the innate immune sensor cGAS, a member of the same protein family.^40^ The structural studies of the pOAS1·dsRNA·25A_2_·PP_i_·Mg_A_^2+^·Mn_B_^2+^ complex reported in this work confirmed that Mn^2+^ can isomorphously replace Mg^2+^ in the catalytic center of the OAS and support product formation. To further explore the role of metal ions in the catalysis of OAS and the specific impact of manganese, we conducted a series of crystallographic and kinetic experiments, complemented by QC calculations of the OAS reaction pathway in the presence of Mn^2+^ ions.

### The Crystal Structure of the Mn^2+^-Bound Prereactive State Complex of pOAS1

The Mn^2+^-bound prereactive state complex was obtained through a two-step crystal soaking procedure. First, native prereactive state pOAS1·dsRNA·ApCpp_2_·Mg_A/B_^2+^ crystals were prepared and then soaked in an EDTA-containing solution to remove Mg^2+^ ions. The crystals were subsequently transferred into a solution containing 2 mM MnCl_2_ and 1 mM ApCpp to facilitate the formation of a new prereactive state complex with Mn^2+^. The crystal structure was solved by molecular replacement using the prereactive state complex (PDB: 4RWN) as a starting model and refined to a high resolution of 1.6 Å. The final electron density was of excellent quality (Table S1), providing well-defined structural details for the entire complex. Particularly strong electron density signals were observed for two Mn^2+^ ions and two ApCpp molecules, allowing their unambiguous positioning at the catalytic center of OAS1 (Figure 5). The overall conformation of the protein and the binding mode for dsRNA remained unchanged compared to the native prereactive complex (Figure 5A).

**Figure 5:**
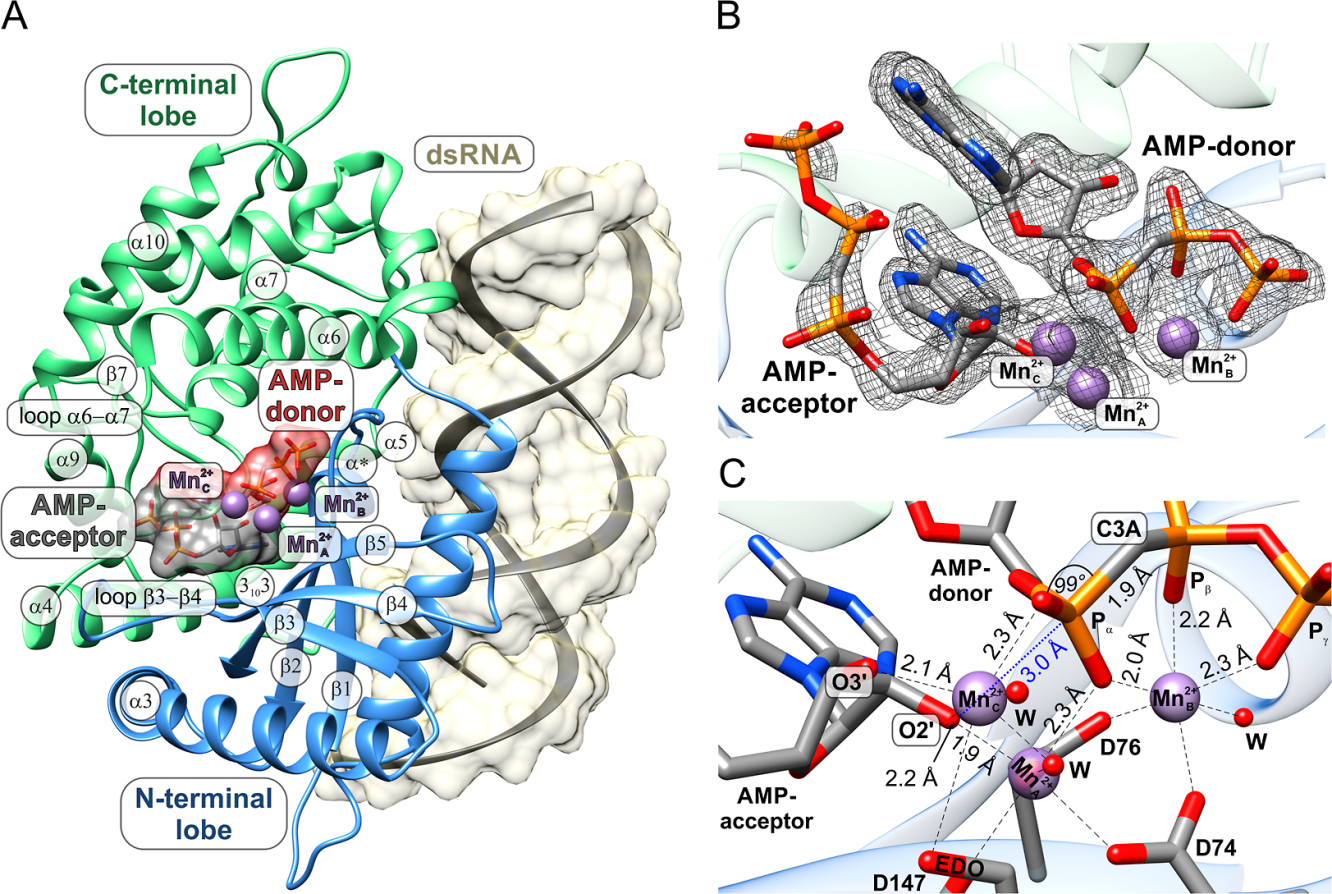
Prereactive pOAS1·dsRNA·ApCpp_2_·Mn_A/B_^2+^·Mn_C_^2+^ complex with two active site-bound molecules of nonreactive ATP analogs ApCpp. (A) Overview of the manganese-bound prereactive pOAS1 complex, including the protein, the dsRNA, and two ApCpp molecules (AMP-donor and AMP-acceptor) bound to the catalytic center together with two manganese ions Mn_A/B_^2+^ and one additional manganese ion Mn_C_^2+^. Key secondary structure elements of pOAS1 are labeled nearby. The dsRNA and the substrate ApCpp molecules are shown with a semitransparent surface. (B) Experimental 1.6 Å 2*F*_o_–*F*_c_ omit electron density at the 1.0 σ level for the Mn^2+^-bound catalytic center of the pOAS1 prereactive state. (C) Close-up representation of the metal-coordinated catalytic center of pOAS1 in the Mn^2+^-bound prereactive complex with the catalytic triad residues D74, D76, and D147, showing the geometric properties of the substrates. Red spheres labeled with “W” represent water molecules. The “EDO”-labeled molecule represents ethylene glycol.

The only notable difference was increased flexibility in the β3–β4 loop of pOAS1, particularly in the flipped conformation of the Q121 side chain. In the Mn^2+^-bound prereactive complex, this residue is oriented toward the active site but does not form interactions with the substrates. The catalytic manganese ions have prominent electron density peaks of 12.4 and 13.7 σ for Mn_A_^2+^ and Mn_B_^2+^, respectively. The anomalous density analysis revealed anomalous peaks at both sites A and B, thus confirming the replacement of the Mg_A/B_^2+^ ions with Mn_A/B_^2+^ (Figure S5B).

The manganese ions form coordination bonds with the substrates, catalytic triad residues D74, D76, and D147, and solvent molecules, acquiring a nearly perfect octahedral coordination symmetry. Surprisingly, the electron density analysis revealed a third metal ion, located between the AMP acceptor and donor molecules near the Mn_A_^2+^ ion (Figure 5B,C). Its electron density peak of 7.7 σ (Figure 5B), combined with the anomalous peak at this position (Figure S5B), corresponds to another manganese ion­(Mn_C_^2+^) with a partial occupancy of 60%. Mn_C_^2+^ exhibits a distorted octahedral coordination, interacting with AMP acceptor atoms O2′ and O3′, the P_α_ group of AMP donor, and solvent molecules (water and ethylene glycol) (Figure 5C). Partial crystallographic occupancy and distorted coordination sphere symmetry indicate increased mobility and weaker binding of Mn_C_^2+^.

The *B*-factor analysis indicates stronger manganese-mediated AMP acceptor binding compared to the native prereactive state. The maximum electron density peak of 4.0 σ for the AMP acceptor is located on the O2′ atom. For the AMP donor, the highest density peak of 8.5 σ was found on the P_β_ group, suggesting that its binding remains stronger than that of the AMP acceptor, similar to that of the native complex. The positioning of both ApCpp molecules at the catalytic center of the OAS remains nearly unchanged compared to the native prereactive complex, except for a slight rotation of the AMP acceptor’s P_β_–P_γ_ moiety toward the adenine group of the AMP donor (Figure 5B,C). The AMP acceptor is stabilized by the same interactions as in the native prereactive state complex and participates in a solvent-mediated H-bond network, which involves residues T67 (α*), Q216 (α6), K219 (α6), and E233 (α7). Additionally, a new hydrogen bond is formed between the R125 side chain and the P_α_ group, increasing the number of stabilizing interactions for the AMP acceptor (Figure S10A). The role of R125 was previously identified in the Mn^2+^-bound postreactive state complex of OAS (Figure S3A,S7), and our findings further confirm its involvement in the exchange of reaction components during the OAS catalytic cycle. Furthermore, the AMP acceptor forms two coordination bonds between the O2′ and the O3′ atoms, and the Mn_C_^2+^ ion with bond lengths of 2.1 and 2.2 Å, respectively, which increases its affinity to the catalytic center. As in the native complex, electron density and stereochemistry analyses of the AMP acceptor indicate that its O2′ group is already deprotonated in the Mn^2+^-bound prereactive state.

The interactions of the AMP donor are the same as those in the native structure. At the same time, we observe shorter coordination bonds between the P_α_ group and both manganese ions, suggesting an increased stabilization for the AMP donor’s P_α_ group. The most striking differences in the AMP donor are observed in the geometry of its α-phosphate. The bond angles O5′–P_α_–C3A, O1A–P_α_–C3A, and O2A–P_α_–C3A are significantly reduced from 107.6°, 107.3°, and 109.1° to 95.5°, 99.3°, and 105.9°, respectively (Figures 5C and S10B). This effect results in a visibly more open umbrella conformation of the reactive P_α_ group. The opening of the umbrella is accompanied by the elongation of the covalent bond P_α_–C3A connecting α- and β-phosphates and coordination bonds between the P_β_–P_γ_ moiety and the Mn_B_^2+^ ion. These structural rearrangements shift the catalytic center of the OAS1 structure closer to the transition-state geometry identified in the QM analysis of the dinuclear catalytic model. A quantitative assessment of how these geometric changes influence the reaction barrier would require a dedicated quantum-chemical investigation of the full reaction pathway for the trinuclear metal configuration.

### Kinetic Studies of OAS1 Reaction in the Presence of Mn^2+^ Ions

Our crystallographic experiments reveal significant effects of manganese ions on the catalytic properties of OAS1. To further investigate how replacing Mg^2+^ with Mn^2+^ ions impacts OAS1 activity, we conducted a series of kinetics measurements with pOAS1. Using a liquid chromatography-based approach to monitor the progress of the reaction, we determined the half-maximal activity concentration (AC_50_) value for Mn^2+^ (0.87 mM ± 0.02 mM) to be 39% lower than for Mg^2+^ (1.42 mM ± 0.02 mM), indicating a higher substrate affinity in the presence of Mn^2+^ ions (Figure 6A). Another striking observation was the change in the Hill slope: Under physiological conditions with Mg^2+^ ions, the Hill slope was 1.83 ± 0.11, which is consistent with the two magnesium cofactor ions bound at the active site of the protein in the prereactive state.^14^ In contrast, with Mn^2+^, the Hill slope increased to 3.17 ± 0.38, suggesting the involvement of a third ion in the course of the pOAS1 catalytic reaction. Transient kinetics experiments, which monitored fluorescence changes upon binding of the fluorescent ATP analogue mant-ATP to pOAS1, provided insight into substrate binding rates (Figure 6B). Rapid mixing of mant-ATP and pOAS1 in the presence of 5 mM MgCl_2_ followed by fitting a single exponential equation to the data resulted in an apparent binding rate constant of 43.3 s^–1^ ± 0.3 s^–1^. In contrast, replacing 5 mM MgCl_2_ with 1 mM MnCl_2_ resulted in nearly 9-fold faster binding, with an apparent binding rate constant of 375.6 s^–1^ ± 7.5 s^–1^. It was not possible to conduct the fitting of the data recorded at higher concentrations of Mn^2+^, as the binding reaction was faster than the time resolution limit of the stopped-flow device. The initial delay phase of approximately 5 ms in Figure 6B, potentially due to biological or technical factors, was excluded from the fit.

**Figure 6:**
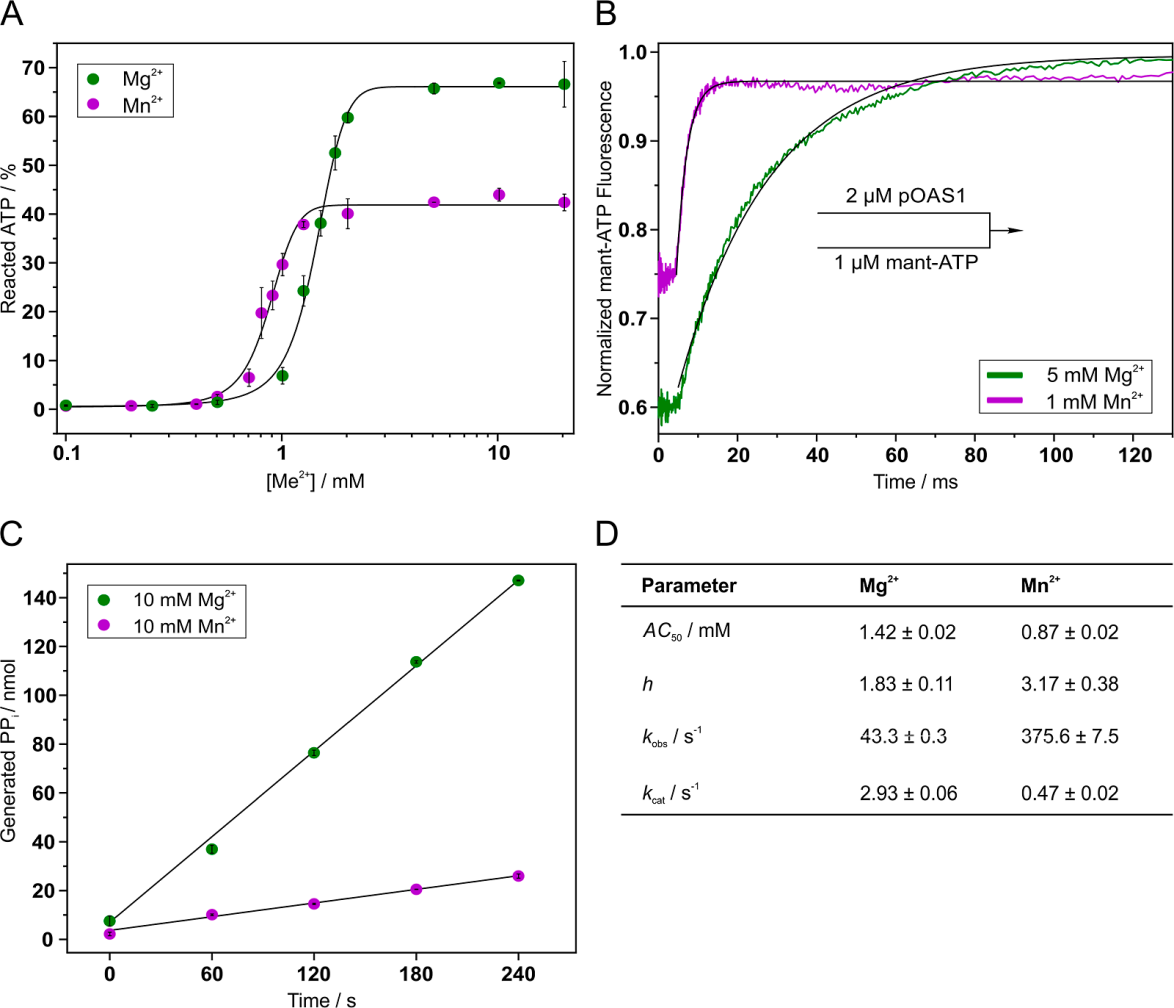
Kinetic analysis of effects of manganese ions on the substrate binding and catalytic reaction of pOAS1. (A) Dose-response curves for the metal ions Mg^2+^ and Mn^2+^ (Me^2+^). Data are the mean of two or three individual experiments ±SD. Four-parameter fits (black curves) were applied to the data, yielding the AC_50_ and Hill slope (*h*) values. (B) Transient-kinetic analysis of pOAS1 substrate binding. The traces present the mean of ten individual measurements. Data were fitted by single-exponential fits (black curves), which allowed one to determine the apparent rates for substrate binding. (C) The activation of pOAS1 by poly­(I/C). Data are the mean of two or three individual experiments ±SD. Data were fitted by linear regression fits (black lines) to calculate the rates of product generation by pOAS1. (D) Compilation of kinetic parameters from (A–C).

We previously demonstrated via a steady state PP_i_ release assay that, with polyinosinic/polycytidylic acid (p­(I/C)) as an activator of OAS1, the *k*_cat_ value for porcine OAS1 is 3.2 s^–1^ ± 0.1 s^–1^.^14^ Repeating this experiment with a liquid chromatography-based approach, where ATP turnover was monitored and PP_i_ production was quantified based on the distribution of 2′-5′-linked products, we obtained a comparable *k*_cat_(Mg^2+^) value of 2.93 s^–1^ ± 0.06 s^–1^ under saturating Mg^2+^ conditions (Figure 6C). Replacing the metal ion with Mn^2+^ resulted in a more than six times slower OAS reaction with *k*_cat_(Mn^2+^) = 0.47 s^–1^ ± 0.02 s^–1^.

### Quantum Chemical Characterization of the OAS Reaction in the Presence of Mn^2+^ Ions

To aid in the interpretation of the experimental structural and kinetic data for the OAS1 reaction in the presence of Mn^2+^ ions, we conducted a QC analysis of the prereactive, transition, and postreactive states along the pOAS1 reaction pathway with two Mn^2+^ ions at positions A and B replacing Mg^2+^ in the catalytic center. The QC studies followed the same methodology used for the native-state calculations. A detailed description of the QC analysis for the OAS1 reaction in the presence of Mn^2+^ ions is provided in the Supporting Information and Materials and Methods sections. The QC analysis supports the crystallographic conclusions that the O2′ group in the Mn^2+^ bound prereactive complex is deprotonated. Our QC calculations reveal that the unpaired 4s 3d electrons of Mn^2+^ form multiorbital bonds with coordinated atoms of OAS1 substrates and protein groups, enhancing interactions between the metal ions and their environment. This increased interaction strengthens the coupling between reacting groups, shifting the prereactive state geometry closer toward the TS (Figure 7A) and significantly enhancing substrate reactivity (Figure 7B). The localization of the spin density peak on the O2′ atom further activates it for the nucleophilic attack through the enhanced electron exchange with α-phosphate. Thus, the 4s 3d electronic shells of manganese play a key role in the additional stabilization of the catalytic center of OAS, the increase of its affinity to the substrates, and its activation for the catalytic reaction. These findings align with our structural (Figure 5C) and transient-kinetic (Figure 6B) data.

**Figure 7:**
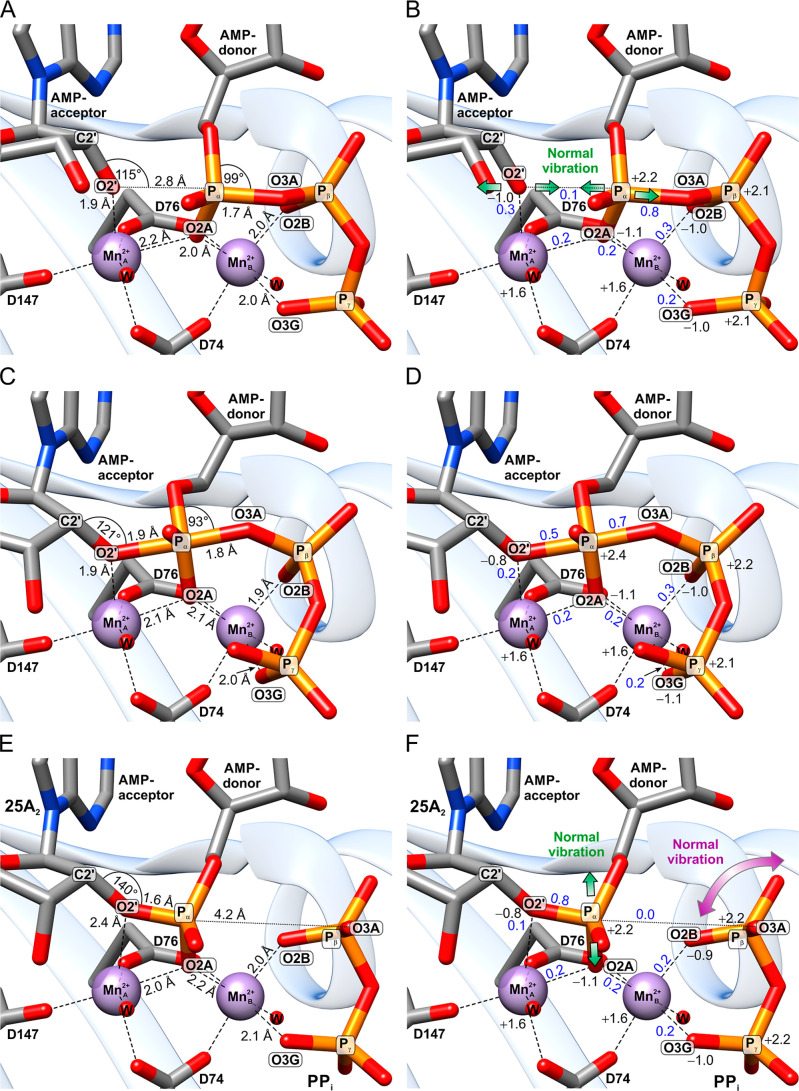
QC-geometry (left column) and electronic properties (right column) of the pOAS1 catalytic center with two Mn^2+^ ions in the prereactive (A,B), transition (C,D), and postreactive (E,F) states. The carbon, nitrogen, oxygen, phosphorus, and manganese atoms are shown in gray, blue, red, orange, and magenta colors, respectively. Hydrogen atoms are omitted for clarity. The DFT NBO charges and bond orders in the right column are shown in black and blue, respectively. The green arrows in panel (B) indicate the QM normal mode, which describes the reaction pathway of OAS. The purple arrow in panel (F) indicates the QM normal mode describing the separation of the P_β_–P_γ_ group from 25A_2_ upon the product formation via the rotational movement of PP_i_. The green arrow in panel (F) indicates the QM normal mode, corresponding to the release of the main product from the catalytic center into the main active site volume.

The formation and stabilization of the Mn^2+^-bound TS are facilitated by the correlation effects and a high spin of 4s 3d electrons of manganese, which lowers the energy barrier of the OAS catalytic reaction. Specifically, the Mn^2+^-bound TS is achieved at a greater distance between the reacting atoms due to enhanced electron exchange along the O2′–P_α_–O3A axis (Figure 7C,D). Similar to the native reaction, the TS formation involves a proton transfer to the P_α_ group of the AMP donor, which contributes to the TS stabilization.

The QC model of the Mn^2+^-bound postreactive state (Figure 7E) closely resembles the native QC geometry (Figure 4E), including protonation of the reactive P_α_ group in 25A_2_ and PP_i_, but exhibits tighter contacts between the Mn^2+^ ions and the reaction products. This stronger binding is facilitated by 4s3d electron exchange effects, including a shift of the spin density maximum onto the PP_i_ moiety. The higher stabilization of products in the Mn^2+^-bound postreactive state (Figure 7F), compared to the native analog (Figure 4F), explains why Mn^2+^ ions enabled the crystallization of the 25A_2_·PP_i_ complex in the catalytic center of OAS1 (Figure 2), whereas all attempts to obtain the equivalent structure in the presence of Mg^2+^ ions were unsuccessful. It also provides insight into the kinetic findings: despite Mn^2+^ increasing substrate affinity and accelerating initial binding (Figure 6A,B), steady-state kinetics indicate reduced OAS1 activity (Figure 6C). This paradox arises because the 4s 3d electrons of Mn^2+^ not only enhance substrate binding but also strengthen product interactions, delaying their release and ultimately inhibiting steady-state turnover.

### Quantum Chemical Characterization of the Trinuclear Metal Center of OAS

To get a deeper insight into how the third metal ion affects the catalytic center, we performed quantum chemical calculations of the prereactive catalytic center of an OAS containing three Mg^2+^ or three Mn^2+^ ions. The starting geometries for the trinuclear metal center were generated with the help of the pOAS1·dsRNA·ApCpp_2_·Mn_A/B_^2+^·Mn_C_^2+^ crystal structure. The initial QC models were subjected to the DFT geometry optimization followed by the NBO and QM-NMA analyses procedures described in the Methods and Supplementary Results sections.

Quantum-chemical characterization of the trinuclear metal ion prereactive states revealed several mechanistic consequences of the transient third metal ion in the catalytic center (Figures S11). The additional ion mediates new stabilizing interactions among the reacting groups, thereby promoting electron exchange among the catalytic components and enabling more favorable proton-transfer pathways. Its presence also enhances the polarization of the phosphate moiety of the AMP donor, increasing both positive and negative charge densities. Notably, the P_α_ becomes more positively charged, rendering an increased susceptibility toward nucleophilic attack. The QM-NMA analysis showed that inclusion of the third metal lowers the vibrational frequency along the reaction coordinate, suggesting facilitated convergence of the reacting groups and a potential reduction in the activation barrier. The third metal ion exhibits a distorted and incomplete coordination sphere compared to ions A and B, which agrees with its weaker binding, partial occupancy, and increased mobility observed in the crystal structure of the Mn^2+^-bound prereactive complex. Finally, the introduction of the third Mn^2+^ ion does not measurably alter the spin density distribution on the reacting atoms compared with the dinuclear Mn^2+^ system.

Since the third metal ion binds only transiently, fully mapping the reaction pathway for the trinuclear catalytic center will require dedicated large-scale quantum-dynamical studies. Nevertheless, quantum-chemical analysis of the prereactive state already highlights several ways in which the third metal ion may enhance the catalytic efficiency of the OAS.

## Discussion

The integration of experimental structural and kinetic data with quantum chemical studies has provided a detailed understanding of substrate binding, catalysis, and product release at the catalytic center of OAS1 (Figure S2). These mechanisms underpin the biological function of OAS1 as an innate immune sensor that generates 2′-5′-oligoadenylates, which activate the RNase L immune pathway in response to pathogen-derived dsRNA. Our findings demonstrate that the geometry, charge distribution, and electronic properties of the OAS1 catalytic center create optimal conditions for the S_N_2 reaction. The nucleophilic attack occurs along a thermally activated low-frequency normal mode, facilitating the convergence of the reacting groups (Figure 4B). As the O2′ of the AMP acceptor approaches the P_α_ of the AMP donor, their movement disrupts the octahedral symmetry of Mg_A_^2+^, reducing its chemical bonding and electrostatic interaction with O2′ (Figure 4D). While the O2′–P_α_ interaction acquires a partial covalent character, the chemical bonding of P_α_ with the leaving group weakens. The further approach of the O2′ and P_α_ atoms leads to the stereochemical Walden inversion of the α-phosphate, the formation of a covalent O2′–P_α_ bond, and the separation of a leaving pyrophosphate group from the newly formed 25A_2_ product (Figures 2 and 4E). The formation of a covalent bond between the AMP donor and acceptor disrupts the coordination bonds of 25A_2_ with magnesium ions, which leads to destabilization of the 25A_2_ position. As a result, weakly bound 25A_2_ dissociates from the catalytic center into the main volume of the active site cavity (Figures 3 and 4F). Brownian motions of the solvent-exposed regions of 25A_2_ may further facilitate its release. Notably, the product dissociation pathway predicted by the QM analysis matches the structural dissociation channel observed in the protein.

The departure of 25A_2_ weakens the PP_i_ interaction with the catalytic center by lowering the magnesium coordination number and disrupting its symmetry, thereby creating favorable conditions for PP_i_ dissociation (Figures 3 and 4G,H). This sequential release of reaction products—25A_2_ first, followed by PP_i_—is governed by the coordination properties of the metal cofactors. This mechanism may be advantageous for subsequent rounds of step-growth oligomerization, ensuring efficient catalytic turnover. Our data rule out a direct relocation of 25A_2_ to the acceptor site at the catalytic center for the subsequent reaction cycle and demonstrate that dissociation of 25A_2_ is a necessary step before the binding of a new substrate binding. The experimentally observed remote binding site for 25A_2_ corresponds to a weakly bound postreactive state complex of the OAS, revealing interactions between the product and the α6–α7 and β3–β4 loops at the exit of the active site. These interactions may play a role in redirecting 25A_2_ back into the catalytic center for the subsequent round of oligomerization. Magnesium ions ensure a catalytic effect of OAS1 through proximity and orientation effects on the reactants, bond strain effects on the P_α_ group of the AMP donor, electrostatic activation of the reacting groups, and stabilization of the TS. The coordination bonds formed by the metal ions and their electronic properties are crucial for both substrate binding and product release. Directed molecular orbital interactions and electron exchange between the metal ions and the reaction components are particularly important in these processes. Thus, the quantum properties of a dinuclear catalytic metal center are central to the immune response activation function of the OAS1.

The high sensitivity of quantum interactions to changes in the geometry of the catalytic center suggests that this property can be exploited in the design of compounds that modulate the activity of the OAS family proteins and related proteins. For example, studies on the mechanism and inhibition of NTase UDP-glucose pyrophosphorylase (UGP) have shown that small perturbations in the sugar-binding loop of UGP can disrupt enzymatic activity by preventing the proper overlap of frontier molecular orbitals between substrates.^34^ A similarly fine-tuned perturbation of the active site of the OAS1 could lead to either inhibition or activation of its catalytic function.

To further explore the relationship between the OAS activity and the electronic properties of the metal cofactors, we investigated the reaction of OAS in the presence of manganese ions using a combination of crystallographic, kinetic, and quantum chemical methods. Substitution of the Mg_A/B_^2+^ with manganese ions at the active site of OAS1 results in an enhanced substrate affinity, binding rate, and chemical activation during the catalytic reaction. These effects originate from the properties of the unpaired 4s3d electrons of manganese, which form multiorbital bonds with the coordinated atoms of substrates and protein groups, strengthening their interactions. Additionally, these bonds increase the interactions between the reacting atoms, shifting the prereactive geometry toward the TS (Figures 4A,B vs [Fig fig7]A,B and 5C). The tendency of 4s 3d electrons to localize on the O2′ atom further activates the reagents for nucleophilic attack through enhanced electron exchange, as indicated by experimental electron density and QM spin density analyses. The correlation effects and a high spin of manganese 4s 3d electrons thus facilitate the formation of the Mn^2+^-bound TS, allowing it to form at a longer distance between the reacting atoms and lowering the energy barrier of the OAS1 catalytic reaction. At the same time, the interactions of 4s 3d electrons also stabilize the postreactive state, which in turn hinders product release, exerting an inhibitory effect on the OAS1 function. The mechanism we describe for the manganese-induced changes in substrate binding, catalysis, and product release provides an explanation for previous findings that metal ion exchange can significantly impact human OAS1 catalysis,^39^ as well as for recent data showing pronounced effects of manganese on the closely related innate immune sensor cGAS.^40^

Our studies underscore the important role of proton transfer in the OAS1 mechanism, facilitating nucleophile activation, TS stabilization, product separation, and dissociation. These conclusions align with the ones from the recent kinetic study of the hOAS1 reaction by R.L. Stein.^33^ Our high-resolution crystallographic and quantum chemical analyses suggest that the O2′ nucleophile is already deprotonated in the prereactive state, indicating early stage deprotonation during complex formation. The general base function is likely fulfilled by an Asp residue in the catalytic triad, a Mg^2+^-coordinated water molecule, or both, mirroring the two-metal-ion mechanism of DNA/RNA polymerases, where metal ions and conserved aspartates coordinate proton transfer and nucleophilic attack.^41^ TS formation involves proton transfer to the P_α_ group of the AMP donor. This proton helps stabilize the TS and is retained in the main product, as supported by the QC analysis. Additionally, lysin 212 in pOAS1 functions as a general acid, transferring a proton to the PP_i_ moiety and facilitating its separation from the main product. The resulting protonation states of 25A_2_ and PP_i_ weaken the electrostatic interactions of the products with the catalytic center, thus promoting their further dissociation. These mechanisms remain unchanged when the catalytic Mg^2+^ ions are replaced by Mn^2+^. At the same time, many aspects of proton transfer in the OAS reaction remain unresolved and require further experimental and theoretical investigations.

Our structural and kinetic studies revealed the potential involvement of a third metal ion, Mg_C_^2+^, in the OAS1 catalysis (Figures 5 and 6A). Until now, OAS1 has been understood to follow a two-metal-ion mechanism for generating second messengers,^14,42,43^ and the presence of a third metal ion has never been observed before in the catalytic center of either OAS or cGAS family proteins. However, in recent years, several studies have reported the presence of third metal ions in the active sites of other NTases with two-metal-ion catalytic centers.^44^ In particular, essential enzymes, such as topoisomerases,^45^ nucleases,^46–48^ and polymerases,^49–53^ have been observed to recruit a third metal ion for catalysis. Although the third metal ion displays partial occupancy in the crystal structure of pOAS1 prereactive complex, a well-defined electron density, a significant anomalous signal, chemically justified coordination, and a below-average *B*-factor argue against a crystallographic artifact. The Mn^2+^-dependent Hill coefficient of 3.17 ± 0.38 further supports the participation of more than two divalent ions in catalysis. Together with the established three-metal configurations in related NTases, these observations are consistent with the functional role of the third metal ion in OAS1 catalysis. Structural comparison of pOAS1·dsRNA·ApCpp_2_·Mn_A/B_^2+^·Mn_C_^2+^ complex with the prereactive state of cGAS (PDB: 7UXW)^40^ (Figure S12) shows that cGAS can also form a trinuclear catalytic metal center and involve a third metal ion in its activity.

Our data suggest that the binding of a third metal ion to the catalytic center of OAS1 is relatively weak and transient due to its distorted and weakly coordinated environment. This finding offers an explanation for the absence of detection of this third metal ion in previous studies. Our QC studies demonstrate that a third metal ion can modulate the catalytic landscape by enhancing electrostatic activation of the substrate, improving alignment of the reacting groups, and potentially lowering the barrier for bond formation. These findings suggest that transient trinuclear configurations may facilitate functionally relevant catalytic states in the OAS and possibly in cGAS, warranting further investigation using experimental and full-quantum-dynamics approaches.

The transient metal binding site C presents a promising target for small molecules that modulate the OAS activity. While the exact functional significance of this third metal ion in OAS1 remains unclear, its presence in the catalytic center of an innate immune sensor suggests that the recruitment of transient ions as cofactors is a common feature among NTases with a dinuclear metal center.

NTases are ancient enzymes central to various biological processes,^54,55^ including DNA replication and repair,^56,57^ transcription,^58^ RNA processing,^59–61^ viral replication,^62,63^ and signal transduction, as exemplified by OAS and cGAS family members.^14^ It can thus be concluded that the evolution of these enzymes must have taken place at the earliest stages of life with the ancestral NTase likely possessing broad and versatile functions. The “minimal” NTases (MNTs) in archaea (e.g., *S. solfataricus* ^64^) and bacteria (e.g., *L. pneumophila* ^65^) provide insight into the early forms of this enzyme family (Figure S13).^54^ These small enzymes, consisting of up to 120 residues, are simple and promiscuous,^54,66,67^ consisting of one rigid catalytic domain coordinating two essential divalent metal ions.^55^ The MNT fold is present in all modern NTases.^54,66^ The catalytic centers of the OAS family proteins share significant similarities with those of other NTases, including cGAS and polynucleotide polymerases. These similarities suggest that the quantum properties of the dinuclear metal center are crucial for the function of these evolutionarily related enzymes, while their differences in specificity and cellular interactions arise from additional structural elements acquired later in the evolution. For example, the structural variations outside the catalytic center in OAS and cGAS family proteins are responsible for their functional differentiation, including activation of distinct immune pathways, as previously described.^14^

In summary, this study reveals the geometry of the OAS1 after product formation and dissociation, the sequential order of product release, and the central role of metal ions in catalysis. It also uncovers the electronic mechanisms underlying the OAS1 activity and highlights their crucial role in its immune-activating function. Notably, we identified a third metal ion in the OAS1 catalytic center with a potential role in catalysis. Substituting Mg^2+^ with Mn^2+^ ions increased the substrate binding rate 9-fold and enhanced substrate activation for catalysis. These findings have broad implications not only for the characterization of OAS but also for the larger family of evolutionarily related NTases, which are essential to numerous biological processes. The insights gained from this study can inform new strategies for regulating these enzymes and to deepen our understanding of their mechanisms. Furthermore, our work emphasizes the importance of QM electronic effects in biological processes, including evolution.

## Methods

### Protein Production

Porcine OAS1 (pOAS1) wild-type (WT) (UniProt ID: Q29599) containing a C-terminal His_6_-tag was cloned in the pET-21a­(+) bacterial expression vector (BioCat). The plasmid encoding the pOAS1 R125A mutant with a C-terminal His-tag optimized for *E. coli* expression was purchased from BioCat. Proteins were prepared based on the procedure described by Torralba et al.^68^ The detailed protocols are presented in the Supplementary Methods part of Supporting Information.

### Crystallization Procedures

For producing the 25A_2_-bound and the postreactive crystals, protein–RNA complexes were prepared at 4 °C by mixing the components to final concentrations of 8.5 mgmL^–1^ protein, 2.5 mgmL^–1^ dsRNA, 0.5 mM ApCpp (Jena Bioscience), and 1 mM NaOH in crystallization buffer (10 mM HEPES pH 7.2, 330 mM NaCl, 10 mM MgCl_2_, 2 mM DTT, 3% (v/v) Glycerol). For crystallization, 19 bp dsRNA (OligoFactory, USA, one strand: 5′-GGCUUUUGACCUUUAUGAA-3′, annealed to its reverse complementary strand) was used, which was previously shown to be a potent activator for OAS.^14,69^ After 30 min on ice, the mixture was spun down for 10 min at 21,000 rcf. For crystallization, the supernatant was mixed in a 1:1 ratio with reservoir solution (100 mM *Tris* HCl pH 7.6, 33–34% (v/v) PEG200) into droplets of 3 μL each. Crystallization was carried out in a hanging drop vapor diffusion setup against 300 μL of a reservoir solution at 18 °C. After crystals had formed and grown to about 500 × 50 × 50 μm^3^, they underwent soaking in cryo-protective solution (100 mM *Tris* HCl pH 7.6, 50% (v/v) PEG200) as follows: (a) 25A_2_-bound postreactive state crystals: soaking overnight in cryo solution incl. 1 mM MnCl_2_ and 20 mM ATP (Sigma-Aldrich); (b) Mn^2+^-bound prereactive state crystals: soaking overnight in cryo solution incl. 2 mM EDTA, then soaking overnight in cryo solution incl. 2 mM MnCl_2_ and 1 mM ApCpp, in between washing the crystals with cryo solution to remove the EDTA–Mg^2+^complexes. Soaking took place in a hanging drop (crystals in 4 μL of cryo solution, supplemented as described above) vapor diffusion setup against 300 μL of cryo solution. After soaking procedures, the crystals were flash-frozen and stored in liquid nitrogen.

25A_2_-dissociated crystals were produced by mixing 1.5 μL of the protein complex (1.9 mgmL^–1^ protein, 0.4 mgmL^–1^ 19 bp dsRNA, as above, 0.5 mM mant-ATP (Jena Bioscience), 0.9 mM NaOH in crystallization buffer, 10 min on ice, spun down for 10 min at 21,000 rcf) in a 1:1 ratio with reservoir solution containing 50 mM *Tris* HCl pH 8.3, 100 mM KCl, 10 mM MgCl_2_, 23% (v/v) PEG400. Crystallization was carried out in a hanging drop vapor diffusion setup against 500 μL of reservoir solution at 18 °C. Crystals were transferred into a cryo-protective solution consisting of 100 mM *Tris* HCl, pH 8.0, and 50% (v/v) PEG200, flash-frozen, and stored in liquid nitrogen.

### Diffraction Data Collection, Processing, and Structure Determination

The X-ray diffraction data for all complex crystals were collected at the PETRA III beamline P11, DESY, Hamburg. The data were integrated with XDS^70^ and scaled with SADABS (Bruker, 2013), APEX2, SADABS (Version 2.03), SAINT-Plus, and XPREP, Bruker AXS Inc., Madison, Wisconsin, USA program packages. Molecular replacement was performed with Phaser^71^ of the CCP4 suite.^72,73^ The previously published model of the pOAS1 prereactive complex (PDB: 4RWN) was used as a search model. The refinement was performed with Refmac5,^74^ ARP/wARP solvent,^75^ and Coot.^76^ To help resolve flexible parts of the structure, the solvent flattening and histogram matching phase improvement procedures in Parrot^77^ (CCP4) were applied using Hendrickson–Lattman coefficients resulting from the ARP/wARP omit map calculations. These procedures helped improve the electron density and increase the signal-to-noise ratio in the weak regions. The anomalous density analysis for the manganese ions was performed with the Phenix program package.^78^ The final electron density maps were of excellent overall quality, and the models had good stereochemistry and low coordinate errors (Table S1).

### Structure Analysis and Figure Generation

Structural analyses were performed by using Coot. Structural comparisons were realized by superimposing the corresponding models using the SSM tool^79^ in Coot. The volume and inner surface area of the active site cavity were calculated using the CASTpFold server.^80^ Molecular graphics were prepared with UCSF Chimera v1.16,^81–84^ developed by the Resource for Biocomputing, Visualization, and Informatics at the University of California, San Francisco, with support from NIH P41-GM103311. Figures were prepared by using CorelDRAW Graphics Suite X6.

### Steady-State Kinetics

Steady-state measurements for pOAS1 WT and activity tests for R125A mutant were carried out based on previously published data.^85–87^ The detailed protocols are presented in the Supplementary Methods part of the Supporting Information.

### Transient Kinetics

Effects of manganese ions on the substrate binding of OAS1 were tested by transient kinetics measurements using a HiTech Scientific SF61 stopped-flow system (TgK Scientific Limited, Bradford on Avon, UK) at 25 °C essentially as described previously.^14,88^ In brief, a 1:1 fast mixing of 2 μM pOAS1 and 1 μM mant-ATP (Jena Bioscience) was performed in a buffer solution containing 20 mM *Tris* pH 7.4 and 10% (v/v) Glycerol, supplemented by 5 mM MgCl_2_ or 1 mM MnCl_2_, respectively. Mant-fluorescence was excited at 365 nm and monitored through a KV389 cutoff filter. For analysis, data were preprocessed with Kinetic Studio v2.0.34.18,495 (TgK Scientific) before being exported to OriginPro (OriginPro v2024, OriginLab Corporation, Northampton, MA, USA). Then, data were fitted by single-exponential fits, which allowed determination of the apparent rates for substrate binding.

### Quantum Chemical Studies of the OAS1 Reaction

The Quantum Chemical characterization of geometries and electronic properties of the OAS catalytic center along the reaction pathway was performed using the Firefly Quantum Chemistry package,^89^ partially based on the GAMESS (US)^90^ source code. All quantum chemical calculations were performed on the “Halime” high-performance computing (HPC) cluster at Hannover Medical School. The cluster consists of dedicated login and compute nodes interconnected via an InfiniBand network and includes GPU-accelerated nodes equipped with NVIDIA Tesla P100 GPUs, as well as parallel BeeGFS and NVMe storage systems. The available computational resources enabled the efficient execution of the ab initio calculations employed in this study.

The QM subsystem of the QC models (103–110 atoms) included the OAS substrates or products, metal ions, protein groups coordinating the substrates and cofactors, and water molecules in the coordination spheres of metal ions. The rest of the protein environment was included in the MM part using the MM3 force field parameters.^91–93^ Steric effects imposed on the catalytic center by the protein environment were modeled via a system of constraints on the atomic positions.

The structures along the OAS1 reaction pathway were optimized by using a QC geometry optimization procedure. All geometry optimizations, Hessian calculations, and QM-NMA analysis, saddle point location, and Intrinsic Reaction Coordinate (IRC) calculations were performed using the DFT with B3LYP hybrid functional,^94,95^ Vosko–Wilke–Nusair electron gas formula 5 correlation^96,97^ and a triple-ζ valence basis set (TZV) for the QM atoms. The QM-NMA of the pre- and postreactive states was performed via the DFT Hessian calculations. The electronic properties of the catalytic center were analyzed using the NBO analysis^98^ based on the DFT wave function.

The employment of the B3LYP hybrid functional ensured a balanced treatment of exchange and correlation effects in the multinuclear-metal-containing catalytic center. In particular, the description of quasi-degenerate Mn 4s 3d states and reaction barrier energetics required an accurate account of the electron correlation. Cartesian Gaussian basis functions were used throughout (no transformation to spherical harmonic functions was applied). The exchange–correlation potential was evaluated using atom-centered numerical quadrature grids consisting of 63 radial shells combined with Lebedev angular grids of order 29 (302 angular points per shell). The Mura–Knowles radial scheme and Stratmann–Scuseria–Frisch partitioning were employed, together with standard grid pruning. Basis function contributions below ∼10^–10^ and grid weights below ∼10^–20^ were neglected. Default initial level shifting of the virtual orbital space was applied to enhance the self-consistent field (SCF) stability; no additional user-defined level shifting was introduced.

The SCF procedure employed the direct evaluation of two-electron integrals with DIIS acceleration. Convergence was assumed when the change in total energy between successive iterations was below 10^–9^ Hartree and the density matrix change was below 10^–5^, with a DIIS error threshold of 10^–7^ Hartree. Two-electron integrals were screened using the Schwarz inequality with a threshold of approximately 10^–9^, while primitive Gaussian products below ∼10^–20^ were neglected.

Geometry optimizations were carried out in delocalized internal coordinates using a quasi-Newton quadratic approximation algorithm. The convergence criterion for the gradient norm was 10^–4^ (a.u.), with corresponding root-mean-square and maximum gradient thresholds of approximately 3 × 10^–4^ and 5 × 10^–4^, respectively, and an energy change criterion of 10^–5^ Hartree.

The generation of QC models for each state along the OAS reaction pathway is described in the corresponding parts of the Supplemental Results section of the Supporting Information. The TS geometry was located at a point with a single imaginary frequency of the Hessian using a quadratic approximation augmented Hessian technique. For obtaining the QC model of the native postreactive state of OAS1, the steepest descent path in mass-weighted Cartesian coordinates using the IRC method was used.^99^ The free energy barriers for forward and backward reactions were calculated by using the harmonic normal mode approximation.

## Supplementary Material

















## Notes

Detailed quantum chemical studies of the OAS1 reaction and postreactive states in the presence of Mg^2+^ or Mn^2+^ ions, along with the description of protein production, steady-state kinetics, and the conversion of OAS product distribution into the amount of generated PP_i_; details of MQD simulations of 25A_2_ dissociation; crystallographic data and refinement statistics for the structures of pOAS1 complexes; mechanism of multistep nucleotidyl transfer in the formation of 2′-5′-linked oligoadenylates by OAS1; enzymatic cycle of OAS1; superposition of key structural elements from the prereactive and postreactive pOAS1·dsRNA·25A_2_·PP_i_·Mg_A_^2+^·Mn_B_^2+^ complexes; assignment of secondary structure elements to the amino acid sequence of pOAS1; anomalous density of metal binding sites in the three complexes; interactions of AMP donor and acceptor nucleosides in the postreactive OAS1 complex; relative amount oligoadenylate products synthesized by pOAS1 WT or R125A mutant; stabilization of the PPi byproduct in the postreactive OAS1 complex; superposition of the postreactive complexes; hydrogen bond between R125 and the AMP acceptor; QC geometry and electronic properties of trinuclear Mg^2+^ and Mn^2+^ catalytic center pOAS1 in the prereactive state; representative chromatograms from the pOAS1 activity test; and supplementary references (PDF)
pOAS1 prereactive Mg QC model (PDB)
pOAS1 TS Mg QC model (PDB)
pOAS1 postreactive Mg QC model (PDB)
pOAS1 postreactive 25A_2_ dissociated Mg QC model (PDB)
pOAS1 prereactive Mn QC model (PDB)
pOAS1 TS Mn QC model (PDB)
pOAS1 postreactive Mn QC model (PDB)
