# Supplementary material for: The Enzymatic Mechanism of OAS: How Metal Ions and Quantum Effects Help Activate Innate Immunity
Source: ACS Omega. 2026 Apr 13;11(16):24250–68. doi: 10.1021/acsomega.5c13236 (PMC13129866; doi:10.1021/acsomega.5c13236)
Supplement: Supplementary file 1 [file ao5c13236_si_001.pdf]

Supporting Information

The Enzymatic Mechanism of OAS: How Metal Ions and Quantum Effects Help Activate Innate Immunity

Pavel Kats[1,§], Xiaoyi Zhou[1,§], Jannik Wiebe[1], Ole Zeymer[1], Petra Baruch[2], Manuel H. Taft[1], Patrick Y. A. Reinke[3], Sebastian Günther[3], Alke Meents[3], Rune Hartmann[4], Dietmar J. Manstein[1,2], Roman Fedorov[1,2*]

[1]Institute for Biophysical Chemistry, Fritz–Hartmann–Centre for Medical Research, Hannover Medical School, Carl-Neuberg-Strasse 1, 30625 Hannover, Germany

[2]Reseach Division for Structural Biochemistry, Hannover Medical School, Carl-Neuberg-Strasse 1, 30625 Hannover, Germany

[3]Center for Free-Electron Laser Science CFEL, Deutsches Elektronen-Synchrotron DESY, Notkestrasse 85, 22607 Hamburg, Germany

[4]Department of Molecular Biology and Genetics, Aarhus University, Universitetsbyen 81, 8000 Aarhus, Denmark

[§]equally contributing

[*]Correspondence should be addressed to: Fedorov.Roman@mh-hannover.de

Table of Contents

Supplementary Results S3

Supplementary Methods S12

Supplementary Tables S16
  Table S1 .... S16

Supplementary Figures S17
  Figure S1 .... S17
  Figure S2 .... S18
  Figure S3 .... S19
  Figure S4 .... S20
  Figure S5 .... S21
  Figure S6 .... S22
  Figure S7 .... S23
  Figure S8 .... S24
  Figure S9 .... S25
  Figure S10 .... S26
  Figure S11 .... S27
  Figure S12 .... S28
  Figure S13 .... S29
  Figure S14 .... S30
Supplementary References S31

Supplementary Results

Computational studies of OAS1 reaction and post-reactive states

The pre-reactive state characteristics

The initial geometry for the QC study was derived from the high-resolution structure of the pre-reactive state of pOAS1 (PDB-ID: 4RWN) [1]. In this state, the main interaction of the AMP acceptor with the catalytic center is the coordination bond between the nucleophile O2' atom of the ribose and the cofactor Mg_A^{2+}. As demonstrated by the study of Donovan *et al.* [2], the lack of the O2' group in dATP leads to a loss of the AMP acceptor binding to the catalytic center of OAS1. The QC analysis and high-resolution X-ray crystallographic structures of pOAS1 pre-reactive complexes (PDB-ID: 4RWN, 9NYB, and unpublished data) show that the O2' group is already deprotonated in the pre-reactive complex (see the "Quantum chemical studies of OAS1 reaction and post-reactive states" section in the main text). Its artificial protonation, followed by a subsequent QC geometry optimization, renders an altered binding mode of the AMP acceptor that is inconsistent with the experimental structures.

The cofactor ions Mg_A^{2+} and Mg_B^{2+} are coordinated by the residues D74, D76, and D147 of the catalytic triad. They have octahedral coordination spheres occupied with carboxylate oxygens of the catalytic triad, ribose and phosphate oxygens of the substrates, and water molecules (Figure 4A).

As demonstrated in our previous study [1], in the pre-reactive state, the reactive groups O2' (AMP acceptor) and P_α–O3A (AMP donor) are aligned on the same axis, which forms the S_N2 reaction coordinate. The O2' atom is positioned 3.0 Å away from P_α at sufficient proximity to allow for the overlap of frontier molecular orbitals, electron exchange, and the start of a nucleophilic substitution reaction. The positions of the reacting atoms are stabilized by the coordination bonds with magnesium cofactors, which play an essential role in the proximity and orientation effect of OAS1 catalysis. O2' is located at an axial position of the Mg_A^{2+} coordination sphere, with an O2'–Mg_A^{2+} distance of 2.0 Å. The angle between the O2'–Mg_A^{2+} coordination bond and the equatorial plane of the Mg_A^{2+} coordination sphere is very close to 90° as in nearly perfect octahedral geometry. The P_α group of the AMP donor is stabilized by the 2.0 Å coordination bond O2A–Mg_B^{2+} (Figure 4A), and 2.5 Å contact with Mg_A^{2+}. The interaction of O2A with Mg_B^{2+} imposes a flattening effect on the P_α group geometry, which renders it closer to the transition state. Thus, the magnesium cofactor has a bond strain activation effect on the reacting group. The P_β and P_γ groups of the AMP donor are stabilized by the coordination bonds O2B–Mg_B^{2+} (2.0 Å) and O3G-Mg_B^{2+} (2.1 Å), respectively.

The NBO analysis of the DFT wave function provided important insights into the charge distribution and chemical properties of these interactions (Figure 4B). Both magnesium ions with an equal positive charge (+1.8) make electrostatic interactions with the AMP donor oxygen atoms O2A (−1.3), O2B (−1.2), and O3G (−1.3), and the AMP acceptor's O2' (−1.0). The P_α, P_β, and P_γ atoms of the AMP donor carry similar charges between +2.3 and +2.4.
The electrostatic interaction of Mg_A^{2+} with O2' of the AMP acceptor is weaker than the Mg_B^{2+} interactions with the phosphate oxygens of the AMP donor. The O2A atom of the P_α group has a bridging location between Mg_A^{2+} and Mg_B^{2+} and makes electrostatic interactions with both ions. The charge difference between the reacting atoms O2' and P_α contributes to creating a favorable condition for the nucleophilic attack. The polarity of the reacting atoms is increased by the magnesium cofactors. The comparison of the DFT NBO charges on O2' and P_α in the presence and the absence of Mg_A^{2+} and Mg_B^{2+} shows that the magnesium ions increase the negative charge on O2' and the positive charge on P_α, which leads to a nearly twofold increase in the electrostatic interaction energy between the reacting atoms. Thus, the magnesium cofactors have an electrostatic activation effect on the reacting groups.
The NBO analysis of the Mayer-Mulliken bond order matrix revealed some interesting differences in the quantum properties of atomic interactions in the catalytic center. While most coordination bonds responsible for the positioning of the reacting groups have a partly covalent nature (Figure 4B), the interactions between the α-phosphate group of the AMP donor and magnesium ions have zero bond orders. The latter indicates that the α-phosphate is more labile than the P_β–P_γ groups, which is confirmed by the experimental electron density peaks of 8.1 σ on P_α vs 9.0 σ peaks on P_β–P_γ in the crystal structure of the pre-reactive state. It is interesting to note that this phenomenon becomes apparent only at the levels of electron density and quantum mechanical analyses. Stereochemical analysis suggests that the zero order of the O2A–Mg_B^{2+} bond is likely to be due to a distortion of the octahedral symmetry of Mg_B^{2+} coordination sphere. In particular, the angle between the O2A–Mg_B^{2+} axis and the equatorial plane of the Mg_B^{2+} coordination sphere (which contains O2B, O3B atoms, and carboxylate oxygens of D74 and D76) deviates by ≈10° from an ideal geometry. This deviation is sufficient to disrupt the overlap of molecular orbitals between the ligand and the central atom.
The zero-order of the O2A–Mg_B^{2+} bond may have functional meaning for the catalytic reaction since the P_α group should have a certain degree of freedom for its movement towards the AMP acceptor. At the same time, the coordination bond between the nucleophile O2' and Mg_A^{2+} has a bond order of 0.3. This is consistent with the nearly perfect octahedral geometry of Mg_A^{2+} coordination sphere, which ensures an optimal overlap between the molecular orbitals of the coordination center and the ligand.

To obtain insights into the dynamic effects in the pre-reactive catalytic center, we performed the DFT Hessian calculations and quantum mechanical normal mode vibrational analysis (QM-NMA) for the pre-reactive geometry. The QM-NMA provided information about the movements of the atoms and groups in the catalytic center. It should be noted that the QM-NMA was performed for a stationary cluster model within the harmonic approximation; while it characterizes local vibrational modes, it does not by itself provide the full free-energy profile of the reaction or establish dynamical rate control. In particular, it revealed a low-frequency normal vibration, which describes the nucleophilic attack of O2' of the AMP acceptor on P_α of the AMP donor along the S_N2 reaction pathway (Figure 4B). The vibration energy for this mode (0.7 kcal/mol) lies within the thermally excitable range. The large amplitude movement of O2' towards P_α is coupled with a smaller amplitude movement of P_α towards O2' along the O2'–P_α–O3A axis. The closest distance between the reacting atoms along this vibration is 2.6 Å, which increases the overlap of the frontier molecular orbitals and the electron exchange between the reagents.

The transition state characteristics

The pre-reactive state coordinates were used as a starting point for the location of the transition state (TS) geometry. The search for the TS was performed by successively decreasing the distance between the reacting atoms O2' and P_α in increments of 0.1 Å along the O2'–P_α–O3A axis and the low-frequency normal mode described in the previous section. The constrained geometry minimization was performed at each step. Upon reaching the flat conformation of the $P_\alpha O_3$ group of the AMP donor, the precise location search of the TS was performed using a quadratic approximation augmented Hessian technique [3–5]. Transition state optimization was considered converged when the maximum gradient component was below 1.0×10^{-4} a.u., the RMS gradient below ~3×10^{-5} a.u., and the energy change below 1.0×10^{-5} Hartree. The transition state was verified by harmonic vibrational analysis, confirming the presence of exactly one imaginary frequency. The corresponding normal mode was inspected and found to describe the concerted O2′ nucleophilic attack and leaving-group departure along the S_N2 reaction coordinate.

The TS has a characteristic trigonal bipyramidal geometry with equal distances of 1.6 Å for O2'–P_α and P_α–O3A bonds. During the TS formation, both the O2' and the P_α groups move towards each other from their original positions in the pre-reactive state. This movement results in a decrease in the O2A–Mg_A^{2+} distance and an increase in the O2A–Mg_B^{2+} distance, rendering an equidistant bridging position of O2A between the magnesium ions. This position of O2A helps stabilize the flat conformation of the $P_\alpha O_3$ group in the TS. Another factor that helps stabilize the TS is a proton transfer

to the O1A oxygen of the $P_\alpha O_3$ group from the nearest water molecule in the coordination sphere of Mg_A^{2+}. This proton transfer occurs shortly before the flat geometry of the $P_\alpha O_3$ group is achieved. Compared to the pre-reactive state, the chemical bonding between O2' and P_α increases from the bond order 0.0 to 0.5, while the bond order between P_α and the leaving group drops from 0.7 to 0.4 (Figures 4B,D). Thus, the attacking and the leaving groups have nearly identical chemical bonding. At the same time, the movement of O2' towards P_α disrupts the octahedral symmetry of Mg_A^{2+} coordination sphere and increases both the O2'–Mg_A^{2+} distance and the C2'–O2'–P_α angle (Figures 4A,C). These geometric changes lead to a disruption of molecular orbital overlap between O2' and Mg_A^{2+}, the drop of the chemical bonding between these atoms to a zero-bond order, and a decrease of the negative charge on O2'. Together, these factors weaken the O2'–Mg_A^{2+} interaction.

The TS formation is accompanied by a minor conformational change of the leaving P_β–P_γ group and a slight increase in the O2B–Mg_B^{2+} and O3G–Mg_B^{2+} distances. At the same time, the phosphate moiety of the AMP donor becomes more polarized, and the electrostatic charges on phosphorus atoms increase. The bond order increases for the O2B–Mg_B^{2+} and decreases for the O3G–Mg_B^{2+} interactions. Considering the above-mentioned factors, one could conclude that the β-phosphate of the AMP donor becomes more stabilized in the TS compared to the γ-phosphate.

The QC calculations for the dinuclear metal catalytic center predict that the free-energy barrier between the pre-reactive and the transition states is ≈ 21 kcal/mol. The kinetic studies for the pOAS1 reaction performed by Lohöfener *et al.* [1] allow estimating the experimental free energy reaction barrier based on the substrate-to-product conversion value k_{cat} and the Transition State Theory for the S_N2 catalytic mechanism, with a single barrier between the pre- and the post-reactive states. This mapping of k_{cat} to a single free-energy barrier assumes that the chemical transformation step is rate-limiting and that preceding binding or conformational gating processes do not dominate the overall turnover rate. In this case, the free energy barrier can be calculated as $\Delta G^{\#} = -RT \cdot \ln\left(\frac{k_{cat}h}{k_B T}\right)$, where k_B, h, and R are Boltzmann's, Planck's, and ideal gas constants, respectively, and T is the absolute temperature. The experimental k_{cat}-based free energy barrier is ≈ 18 kcal/mol, which is in agreement with the calculated value. Besides, the calculated free energy barrier for the pOAS1 reaction is close to the experimental and the QC calculated $\Delta G^{\#}$ for similar S_N2 reactions of hOAS1 [6], UDP-glucose pyrophosphorylase [7], endonuclease IV [8], and DNA polymerase [9].

The post-reactive state formation and characteristics

The post-reactive state was obtained from the TS geometry using a quadratic approximation augmented Hessian search for stationary points [3–5]. The resulting QC geometry of the post-reactive state is similar to the one observed in the crystal structure pOAS1•dsRNA•25A$_2$•PP$_i$•Mg^{2+}_A•Mn^{2+}_B (Figure 2C). In the post-reactive geometry, the O2' and P_α atoms form a covalent bond, which completes the synthesis of the 25A$_2$ product. The O2'–P_α bond formation causes further dissociation of the O2' atom from Mg^{2+}_A and an increase in the C2'–O2'–P_α angle compared to the TS geometry (Figure 4E). The distances between the O2A atom and both magnesium ions increase while the negative charge on O2A and the energy of its electrostatic interactions with Mg^{2+}_A and Mg^{2+}_B decrease. The Walden inversion causes the reversal of the α-phosphate geometry, which helps separate the pyrophosphate group and sever its chemical bonding with the 25A$_2$ product (Figure 4F). These steric and electronic effects resulting from the O2'–P_α bond formation led to a significant decrease in the interaction energy of the 25A$_2$ reaction product with the catalytic center of OAS1. As a consequence, the QM-NMA analysis of the post-reactive state produces a low-frequency normal mode, corresponding to the release of the main product from the catalytic center into the main active site volume (green arrow in Figure 4F). In this mode, the dissociation of the P_α group from the catalytic center is coupled with the movements of 25A$_2$ parts that are located in the main volume of the active site cavity. These parts of 25A$_2$ are solvent accessible and less stabilized by the protein interactions, which makes their movement likely to happen spontaneously as Brownian motion under physiological conditions. Therefore, after the product is formed and its binding to the catalytic center is weakened (due to the effects described above), the Brownian motions of the solvent-accessible parts of 25A$_2$ could activate the release of the P_α group and whole product into the main volume of the active site.

The separation of the P_β–P_γ group from 25A$_2$ is accomplished via the rotational movement of pyrophosphate around an axis passing through the oxygens O2B and O3G coordinated with Mg^{2+}_B. The rotational movement results in a separation of the leaving O3A atom from the P_α group. At the same time, the positions of the rotation axis oxygens O2B and O3G and their interactions with Mg^{2+}_B ion remain unchanged (Figures 4C, F). The resulting by-product orientation is stabilized by the proton transfer from the active site lysine 212 to PP$_i$, which explains the enhanced PP$_i$-K212 interaction observed in the experimental complex structure. The QM-NMA shows that the rotational separation of PP$_i$ from the AMP donor is described by another low-frequency normal mode shown with a purple arrow in Figure 4F. In contrast to 25A$_2$, the interaction of pyrophosphate with the catalytic center remains quite strong after the formation of the reaction products. It involves the electrostatic interactions and chemical bonding between the oxygens O2B and O3G of PP$_i$ and Mg^{2+}_B ion (with a

combined bond order of 0.5) (Figure 4F). The more stable coordination of PP_i by magnesium in the post-reactive state can explain why it is still bound to the catalytic center in the experimental pOAS1•dsRNA•$25A_2^{diss}$•PP_i•$Mg_{A/B}^{2+}$ complex structure, while $25A_2$ is observed at a dissociated position at the edge of the active site (Figure 3A). To obtain further evidence that the formation of $25A_2$ simultaneously creates favorable conditions for its dissociation from the catalytic center, we performed molecular quantum dynamics (MQD) calculations using a semi-empirical PM3 level of theory. The calculations confirmed that $25A_2$ is less stabilized at the catalytic center than PP_i and dissociates more readily, which fully agrees with the conclusions from the experimental crystallographic and *ab initio* DFT analyses. Besides, the MQD calculations confirmed the QM-NMA indication that the dissociation of $25A_2$ is coupled with the thermal fluctuations of its solvent-exposed parts in the main volume of the active site.

The PP_i state characteristics

The crystal structure of the pOAS1•dsRNA•$25A_2^{diss}$•PP_i•$Mg_{A/B}^{2+}$ complex shows that after the dissociation of $25A_2$ from the catalytic center, the pyrophosphate retains its position and coordination with the Mg_B^{2+} ion (Figure 3E). At the same time, the experimental distances between PP_i and Mg_B^{2+}, as well as the thermal fluctuations of PP_i increase, compared to the crystal structures of pre- and post-reactive complexes (see the description of the pOAS1•dsRNA•$25A_2^{diss}$•PP_i•$Mg_{A/B}^{2+}$ complex structure in the main text). These effects indicate that in the absence of substrates or the main product, the binding of the PP_i moiety to the catalytic center becomes substantially weaker. The DFT calculations and the NBO analysis using the QC model obtained from the experimental pOAS1•dsRNA•$25A_2^{diss}$•PP_i•$Mg_{A/B}^{2+}$ complex structure reveal that both the electrostatic interactions and the bond orders between the PP_i and Mg_B^{2+} are reduced in this state (Figure 4G,H). The weakening of the PP_i binding may facilitate further dissociation of the by-product from the catalytic center. This is supported by the QM-NMA analysis for this state, which produces a low-frequency normal mode corresponding to the exit of the β-phosphate from the coordination sphere of Mg_B^{2+} (purple arrow in Figure 4H).

Quantum chemical characterization of the OAS1 reaction in the presence of Mn^{2+} ions

The QC studies of the OAS1 reaction in the presence of Mn^{2+} ions were performed using the same methodology as for the native state's calculations. The QC model of the manganese-bound dinuclear metal pre-reactive state was generated by replacing $Mg_{A/B}^{2+}$ ions with $Mn_{A/B}^{2+}$ ions in the respective QC

model of the native state and subjecting it to a further quantum mechanical geometry optimization procedure for the high-spin manganese complex (overall multiplicity of 11). The O2' group in the Mn^{2+} bound pre-reactive model was deprotonated, as suggested by crystallographic, stereochemical, and quantum-chemical analyses. The final geometries were used for the NBO and QM-NMA analyses based on the DFT wave function (Figure 7).

The Mn^{2+}-bound pre-reactive state characteristics

The optimized QC geometry of the Mn^{2+}-bound pre-reactive state is similar to the native pre-reactive structure. The main structural differences are localized in the region containing reacting groups, which is in full agreement with the experimental X-ray structural data. The Mn^{2+} ions occupy the same positions as Mg^{2+} and coordinate carboxylate oxygens of the catalytic triad, O2' oxygen of the AMP acceptor ribose, phosphate oxygens of the AMP donor, and water molecules, rendering an octahedral geometry of the metal coordination spheres. The distances between metals and the ligands are somewhat shorter in complex with manganese, indicating stronger interactions compared to magnesium. In particular, the coordination of the P_α group by Mn_A^{2+} via the O2A atom is significantly stronger than in the complex with Mg_A^{2+} (Figures 5A vs 7A), which leads to an increase in the proximity of the reacting groups. The manganese ions also impose a flattening effect on the α-phosphate, rendering its conformation closer to the one in the transition state. The more open conformation of α-phosphate makes the Mn^{2+}-bound pre-reactive state closer to the TS geometry than the native structure. The DFT NBO analysis of electronic properties revealed a significantly higher level of electron exchange between the atoms in the catalytic center in the presence of manganese compared to the magnesium complex. This results in the change of bond orders between the reacting atoms O2' and P_α, and between the O2A and the metal ions from zero to positive values (Figures 4B, 7B), and an overall increase in the correlation energy of the pre-reactive complex by 13%.

The frontier molecular orbital and spin density analyses show that the 4s3d unpaired electrons of manganese are localized mainly on the atoms in the metal coordination sphere. These electrons occupy the first 10 singly occupied frontier molecular orbitals of the catalytic center, all of which are involved in the interactions of Mn_A^{2+} and Mn_B^{2+} with substrates, protein groups, and water molecules. The highest peak of spin density from the 4s3d electrons is located on the O2' atom, which enhances its reactivity through increased exchange interactions, resulting in the positive bond order between the reacting O2' and P_α atoms. The first doubly occupied frontier orbital, lying in energy below the singly occupied 3d orbitals of manganese, is an equivalent of HOMO in the native pre-reactive state, which is localized on the O2' atom and participates in the nucleophilic attack. Together with a larger ionic radius and a

higher electron correlation effect of manganese, the localization of 4s3d unpaired electrons on the atoms of the metal coordination sphere leads to more energy-efficient interactions between the components of the catalytic center, which ensures the activation effect for catalytic reaction and higher affinity of the substrates to OAS1 in the presence of manganese.

The QM-NMA calculations revealed that the normal mode describing the reaction pathway of OAS1 has a lower frequency than the equivalent mode in Mg^{2+}-complex, which provides additional evidence that manganese has a higher activating effect on the OAS1 catalysis than magnesium. It is interesting to note that the electrostatic charges on the reacting groups do not change significantly in the Mn-bound pre-reactive state compared to the native complex, with a tendency to be somewhat lower in the former. This implies that manganese has a lower polarizing effect on the reactive groups and that the higher substrate affinity and activation of the catalytic reaction are achieved mainly due to the quantum properties of the 4s3d electronic shells.

The Mn^{2+}-bound transition state characteristics

The Mn^{2+}-bound dinuclear metal transition state was obtained using the same method as described above for the native TS geometry. The main difference in the TS geometry with manganese is that the saddle point is reached at longer distances between the reacting atoms (Figure 7C). As a result, the O2'–P_α–O3A axis in the trigonal bipyramidal geometry of the Mn^{2+}-bound TS is 0.5 Å longer than in the native structure. The longer O2'–P_α and P_α–O3A bonds in the TS contribute to lowering the reaction barrier to $\Delta G^{\#} \approx 10$ kcal/mol, which is two times lower than in the native TS. Another factor contributing to the lower barrier of the Mn-catalyzed OAS1 reaction is a more efficient stabilization of the TS. Since reaching the TS geometry does not require a large movement of the O2' atom, the octahedral symmetry of the $\mathrm{Mn}_\mathrm{A}^{2+}$ coordination sphere remains essentially undisrupted at the saddle point, which preserves the molecular orbital overlap and a positive bond order between O2' and $\mathrm{Mn}_\mathrm{A}^{2+}$ (Figures 7C,D). The symmetric positioning of O2A between $\mathrm{Mn}_\mathrm{A}^{2+}$ and $\mathrm{Mn}_\mathrm{B}^{2+}$ is geometrically identical to the Mg^{2+}-bound TS. However, the exchange effects of 4s3d electrons result in the positive $\mathrm{Mn}_\mathrm{A}^{2+}$–O2A–$\mathrm{Mn}_\mathrm{B}^{2+}$ bond orders and a higher stabilization of the P_α group (Figures 4C, D, and 7C, D). Proton transfer to the P_α group of the AMP donor occurs by the same mechanism as in the native reaction and contributes to the TS stabilization. Finally, a decrease in the energy gap between HOMO and LUMO in the TS and the localization of the spin density on the reactive atoms enhance the electron exchange along the O2'–P_α–O3A axis by ≈ 33% compared to the native TS (Figures 4D, 7D), which also contributes to a lowering of $\Delta G^{\#}$ and the activation of the catalytic reaction.

The Mn^{2+}-bound post-reactive state characteristics

The Mn^{2+}-bound post-reactive state was obtained using the same method as described above for the magnesium complex. The product formation in the presence of manganese proceeds via the same mechanism as in the native reaction. After the Walden inversion of the α-phosphate, the P_α–O3A bond breaks, and the P_β–P_γ moiety separates from the AMP donor via a rotational movement around the axis, passing through O2B and O3G oxygen atoms. At the same time, the covalent bond between the O2' and P_α atoms is formed (Figure 7E). The resulting post-reactive geometry is nearly identical to its native equivalent, including protonation of the reactive P_α group in $25A_2$ and pyrophosphate. The latter plays a significant role in stabilizing the post-reactive complex. The tendency for manganese ions to make closer contacts with the ligands, observed in the pre-reactive and transition states, persists in the post-reactive geometry, resulting in a tighter binding of the products to the catalytic center. In particular, the bond between Mn_A^{2+} and O2A atom 0.2 Å shorter compared to the Mg_A^{2+}–O2A bond, which provides an additional stabilization of the AMP donor's P_α group in $25A_2$. However, more prominent differences are revealed between the electronic properties of the Mn-bound and native post-reactive states. The bond orders between the P_α group and the metal ions remain positive upon the product formation in the Mn^{2+}-bound complex, while in the native post-reactive state, these interactions have zero bond orders. Even as the O2'–P_α bond formation moves the O2' atom away from Mn_A^{2+} and increases the C2'–O2'–P_α angle, the bond order for this interaction remains in the positive range. These effects reflect an increased electron exchange due to the 4s3d electronic shells of manganese, which provides additional stabilization for the Mn^{2+}-bound products. Additional stabilization of $25A_2$ increases the frequency of the normal mode, describing the dissociation of the main product from the catalytic center (green arrow in Figure 7F).

Upon the product formation, the maximum spin density from the 4s3d electrons shifts from the O2'–P_α area of $25A_2$ to the phosphates of PP_i. The localization of the spin density peak on the by-product renders increased exchange interactions between PP_i and Mn_B^{2+}, which increases the stability of PP_i in the Mn^{2+}-bound post-reactive complex. Similar to the other states along the reaction pathway (Figures 7A-D), the polarizing effect of manganese on the products is lower than that of magnesium, which implies that the quantum properties of the 4s3d electronic shells are mainly responsible for the higher product stabilization at the catalytic center.

Supplementary Methods

Protein production

Proteins were produced in *E. coli* RosettaTM (DE3) pLysS (Novagen) cells in LB medium containing 75 μgmL^{-1} carbenicillin and 20 μgmL^{-1} chloramphenicol for 24 h at 18 °C after induction with 0.5 mM IPTG. Cells were lysed by sonification in lysis buffer (25 mM HEPES pH 7.5, 300 mM NaCl, 20 mM Imidazole, 10 % (v / v) Glycerol, supplemented by 5 mM β-Mercaptoethanol, and protease inhibitor cocktail (Sigma Aldrich)). Purification was carried out using Ni-NTA affinity chromatography with HisTrapTM HP (Cytiva) columns. Proteins were bound to the column in the lysis buffer, then washed with wash buffer A (25 mM HEPES pH 8.0, 500 mM NaCl, 20 mM Imidazole, 10 % (v / v) Glycerol, supplemented by 5 mM β-Mercaptoethanol), wash buffer B (25 mM HEPES pH 6.0, 500 mM NaCl, 20 mM Imidazole, 10 % (v / v) Glycerol, supplemented by 5 mM β-Mercaptoethanol), and wash buffer C (25 mM HEPES pH 6.8, 500 mM NaCl, 20 mM Imidazole, 10 % (v / v) Glycerol, supplemented by 5 mM β-Mercaptoethanol), before being eluted with elution buffer (25 mM HEPES pH 6.8, 500 mM NaCl, 250 mM Imidazole, 10 % (v / v) Glycerol, supplemented by 5 mM β-Mercaptoethanol). After quality control via SDS-PAGE, the cleanest fractions were pooled and dialyzed against dialysis buffer (25 mM HEPES pH 6.8, 330 mM NaCl, 10 mM $MgCl_2$, 1.6 % (v / v) Glycerol, supplemented by 2 mM DTT) overnight, and for four more hours after dialysis buffer exchange. Samples were concentrated, flash-frozen in liquid nitrogen, and stored at −80°C.

Steady-state kinetics

For the activity tests of pOAS1 wild-type and R125A mutant, mixtures consisting of 200 nM protein, 100 $ng\mu L^{-1}$ p(I:C) (Sigma Aldrich), and 1 mM ATP in a buffer solution containing 4 mM Tris-HCl pH 7.5, 15 mM magnesium acetate, 0.2 mM DTT, 20 µM EDTA, 0.1 mg/ml BSA, 2 % Glycerol were incubated for 2 h at 37 °C. Reactions were stopped by heating to 95 °C for 10 min. FastAP Thermosensitive Alkaline Phosphatase (Thermo Scientific) was added to 15 UmL^{-1}. After 2 h at 37 °C, FastAP was heat-inactivated at 95 °C for 10 min. OAS1 products were analyzed using a 1 mL RESOURCE Q column (Cytiva) on an ÄKTA pure 25M (Cytiva) chromatography system. After equilibrating the column with 20 mM Tris-HCL pH 7.5, and spinning down the samples for 10 min at 21,000 rcf, each sample was applied to the column with a flow rate of 0.5 $mLmin^{-1}$. Product separation took place by elution with a linear gradient of 0–35 % 20 mM Tris-HCL pH 7.5, 750 mM NaCl over 17.5 column volumes while monitoring the absorption at 255 nm. Product Analysis was carried out with the "Evaluation Classic" tool of the Unicorn™ Software v7.9 (Cytiva). Peaks in the absorption at λ = 255 nm were integrated with the "Peak Integrate" function, where the baseline was calculated by

the software using the default settings. Separated OAS1 products were assigned according to previously published data [10,11]. Statistical analysis was performed using GraphPad Prism version 8.4.676 for Windows (GraphPad Software, Boston, Massachusetts, USA). $p < 0.05$ was considered statistically significant.

For metal ion-dependent steady-state activity determination, mixtures consisting of 200 nM pOAS1, 100 ngμL^{-1} p(I:C) (Sigma Aldrich), and 1 mM ATP in a buffer solution containing 20 mM Tris pH 7.4 and 10 % (v / v) Glycerol, supplemented by 10 mM $MgCl_2$ or $MnCl_2$, respectively, were incubated for different time periods at 25 °C. All other steps were done in the same way described above, however, on a BIO-RAD NGC chromatography system. Data were exported into Origin 2024, and the areas under the absorption peaks were calculated using the integrate function with default settings. Separated OAS1 products were assigned according to previously published data [10,11]. The product distribution allowed for the calculation of the amount of produced pyrophosphate (see the final formula in the "Conversion of OAS1 product distribution into the amount of generated PP_i" section), which was plotted against the reaction time and analyzed by linear regression in OriginPro (OriginPro v2024, OriginLab Corporation, Northampton, MA, USA).

Dose-response curves were determined by titrating the OAS1 reaction mixtures with different amounts of $MgCl_2$ or $MnCl_2$, respectively. Reactions took place for 1 h at 25 °C, before being stopped by heating to 95 °C for 10 min. All other steps were done as described above. The amount of synthesized pyro-phosphate was plotted against the total concentrations of metal ions, and four-parameter fits were applied in OriginPro (OriginPro v2024, OriginLab Corporation, Northampton, MA, USA) to calculate the maximal response, AC_{50}, and hill slope values.

Conversion of OAS1 product distribution into the amount of generated PP_i

To calculate the amount of PP_i generated by pOAS1 in the course of its enzymatic reaction, we used the product distribution, which was analyzed for each reaction. Since in each pOAS1 reaction, a constant ATP amount of 1 µmol was provided, we can state

$$n_0(\text{ATP}) = 1\ \mu\text{mol} \equiv 100\ \% \quad .$$

The chromatograms show peaks for each component of the OAS1 reaction, which consists of at least one adenosine moiety. This is the case for the ATP substrate and the 25A$_n$ products (see exemplary chromatogram in Figure S14). Calculating the area under each peak, we assume that

$$\sum \int \text{all peaks} \equiv 100\ \% \equiv 1\ \mu\text{mol ATP}$$

and with that

$$\frac{\int \text{ATP}}{\sum \int \text{all peaks}} \equiv \text{ratio of non-reacted ATP}$$

Thus, the ratio r of reacted ATP is

$$r = 1 - \frac{\int \text{ATP}}{\sum \int \text{all peaks}}$$

and we can assume that all products were generated from a total of $n_\text{R}(\text{ATP})$ of ATP, which is

$$n_\text{R}(\text{ATP}) = n_0(\text{ATP}) \cdot r \ .$$

Let's consider the simplest qualitative conversion of ATP to $25A_2$. In such case, all reacted ATP was used by OAS1 to generate the dimeric products, and the peak integration in the respective chromatogram would show:

$$\frac{\int 25A_2}{\sum \int \text{Products}} = 100\ \%$$

The amount of the $25A_2$ product would be $n(25\text{A}_2) = {}^1\!/_2\, n_\text{R}(\text{ATP})$, since one molecule of $25A_2$ is built from two ATP molecules. At the same time, for each generated $25A_2$ molecule, one PP_i molecule is split off from one of the reacting ATPs. With that, the amount $n_\text{G}(\text{PP}_\text{i})$ of PP_i generated in such conversion would be

$$n_\text{G}(\text{PP}_\text{i}) = \frac{n_\text{R}(\text{ATP})}{2}$$

When we consider the conversion of ATP to $25A_2$ and $25A_3$, the chromatogram will show two product peaks, where the ratio β of the dimeric product and the ratio γ of the trimeric product can be calculated as

$$\beta = \frac{\int 25\text{A}_2}{\sum \int \text{Products}} \quad \text{and} \quad \gamma = \frac{\int 25\text{A}_3}{\sum \int \text{Products}} \ .$$

In this case, the amount of the $25A_2$ product would be $n(25\text{A}_2) = {}^1\!/_2\, n_\text{R}(\text{ATP}) \cdot \beta$, and ${}^1\!/_2\, n_\text{R}(\text{ATP}) \cdot \beta$ would also be the amount of PP_i, which emerges from $25A_2$ synthesis. Since one molecule of $25A_3$ consists of three units of ATP molecules, it follows that $n(25\text{A}_3) = {}^1\!/_3\, n_\text{R}(\text{ATP}) \cdot \gamma$. For each generated $25A_3$ molecule, two PP_i molecules have to be released, so the synthesis of $25A_3$

products generates $2 \cdot {}^{1}/_{3}\, n_{\mathrm{R}}(\mathrm{ATP}) \cdot \gamma$ PP_i. With that, the amount $n_{\mathrm{G}}(\mathrm{PP_i})$ of PP_i generated in the whole sample would be

$$n_{\mathrm{G}}(\mathrm{PP_i}) = \frac{\beta \cdot n_{\mathrm{R}}(\mathrm{ATP})}{2} + 2 \cdot \frac{\gamma \cdot n_{\mathrm{R}}(\mathrm{ATP})}{3}$$

When running reactions with pOAS1, we mostly observe the synthesis of up to tetrameric products $25A_4$. Therefore, with the ratio δ of tetrameric products

$$\delta = \frac{\int 25\mathrm{A}_4}{\sum \int \mathrm{Products}}$$

the total amount of generated PP_i is calculated in the respective way:

$$n_{\mathrm{G}}(\mathrm{PP_i}) = \frac{\beta \cdot n_{\mathrm{R}}(\mathrm{ATP})}{2} + 2 \cdot \frac{\gamma \cdot n_{\mathrm{R}}(\mathrm{ATP})}{3} + 3 \cdot \frac{\delta \cdot n_{\mathrm{R}}(\mathrm{ATP})}{4} \quad .$$

Molecular quantum dynamics simulations of $25A_2$ dissociation

The molecular quantum dynamics (MQD) calculations of $25A_2$ dissociation from the catalytic center were performed at the semi-empirical PM3 level of theory implemented in the HyperChem molecular modeling package [12]. The model for the MQD simulations was based on the crystal structure of the post-reactive pOAS1•dsRNA•$25A_2$•PP_i•Mg_A^{2+}•Mn_B^{2+} complex and included structural elements of the pOAS1 active site (described in the "Results" section), two Mg^{2+} ions (Mg_A^{2+} and Mg_B^{2+}), $25A_2$ and PP_i products, and water molecules in the coordination spheres of magnesium. Prior to the MQD simulations, the starting structure was energy-minimized with restrained positions of the protein main chain. The optimized model was subsequently subjected to semiempirical quantum molecular dynamics at a constant temperature of 310 K using a Berendsen weak-coupling thermostat. During the simulations, the positions of the protein backbone atoms were restrained, and the calculations were carried out in the absence of dsRNA.

The MD simulations were performed as a limited number of short, independent trajectories initiated from the optimized post-reactive structure to evaluate whether spontaneous product dissociation follows the direction predicted by the ab initio quantum normal mode analysis. The purpose of these simulations was not exhaustive configurational sampling, but rather to assess the qualitative dynamical feasibility and reproducibility of the dissociation pathway. In all trajectories, product release occurred along the direction suggested by the normal mode analysis, providing consistent mechanistic support for the proposed model.

Supplementary Tables

Table S1: Crystallographic Data and Refinement Statistics for the Structures of pOAS1 Complexes.

	**pOAS1•dsRNA•25A$_2$•PP$_i$•Mg$^{2+}_A$•Mn$^{2+}_B$**	**pOAS1•dsRNA•25A$_2^{diss}$•PP$_i$•Mg$^{2+}_{A/B}$**	**pOAS1•dsRNA•ApCpp$_2$•Mn$^{2+}_{A/B}$•Mn$^{2+}_C$**
PDB code	9NXS	9NY9	9NYB
		Data Collection	
Beamline	P11, DESY	P11, DESY	P11, DESY
Wavelength / Å	1.03323	1.0332	1.03323
Space group	P4$_3$2$_1$2	P4$_3$2$_1$2	P4$_3$2$_1$2
Unit cell parameters:			
a; *b*; *c* / Å	72.1; 72.1; 207.2	71.4; 71.4; 203.6	72.0; 72.0; 206.3
α; *β*; *γ* / °	90.0; 90.0; 90.0	90.0; 90.0; 90.0	90.0; 90.0; 90.0
Resolution range / Å [a]	45.7-1.8 (1.9-1.8)	49.0-2.7 (2.8-2.7)	45.7-1.6 (1.7-1.6)
Crystal mosaicity / °	0.118 [b]	0.100 [b]	0.052
Wilson *B*-factor	39.2 [b]	60.9 [b]	35.7
Unique reflections	51,650 (7,571)	14649 (1448)	72,726 (11,850)
Multiplicity	52.9 (53.7)	13.4 (13.6)	26.4 (24.9)
$\langle I/\sigma(I)\rangle$	22.6 (2.6)	9.1 (2.1)	23.4 (2.2)
Completeness / %	99.8 (99.7)	99.8 (99.7)	99.9 (99.7)
R_{sigma} / % [c]	2.3 (42.4)	7.3 (49.4)	1.9 (41.5)
R_{int} / % [c]	10.2 (88.5)	16.6 (73.9)	5.6 (80.3)
$CC_{1/2}$ / %	100.0 (87.2) [b]	99.8 (94.6) [b]	100.0 (95.2)
		Structure Refinement	
R_{work} / %	19.6	23.4	15.3
R_{free} / %	22.1	25.4	21.1
№ non-hydrogen atoms	4,360	3,907	4,609
№ protein residues	349	349	349
№ RNA bases	38	38	38
№ water molecules	764	205	915
		Geometry	
Coordinate error / Å	0.12	0.37	0.09
r.m.s.d.(bond lengths) / Å	0.007	0.012	0.038
r.m.s.d.(bond angles) / °	1.612	0.788	1.595
$B_{average}$ / Å^2	40.6	69.0	35.6

Statistics for the highest-resolution shell are shown in parentheses. *R.m.s.d.*, root mean square deviation.

[a]High-resolution cutoffs were applied to resolution shells that had the average error in the intensities R_{sigma} > 50 % or had sustained high radiation damage.

[b]Average across data batches.

[c]$\boldsymbol{R}_{\text{sigma}} = \frac{\Sigma[\sigma(\boldsymbol{F}_o^2)]}{\Sigma[\boldsymbol{F}_o^2]}$; $\boldsymbol{R}_{\text{int}} = \frac{\Sigma|\boldsymbol{F}_o^2 - \boldsymbol{F}_o^2(\mathbf{mean})|}{\Sigma[\boldsymbol{F}_o^2]}$

Supplementary Figures

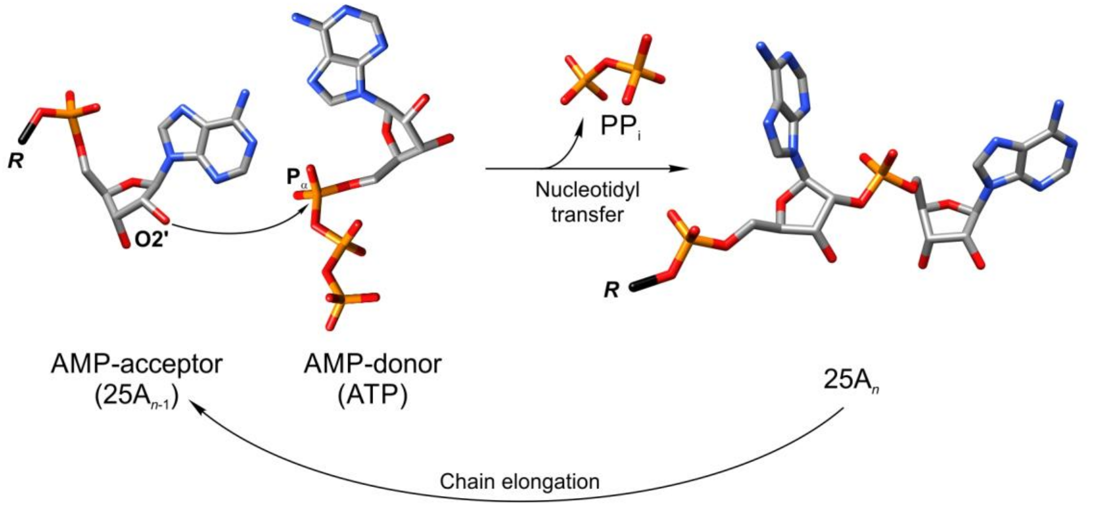

Figure S1: Mechanism of multistep nucleotdidyl transfer in the formation of 2'-5'-linked oligoadenylates by OAS1. Nucleotide transfer takes place via the S_N2 reaction between the AMP-acceptor ($25A_{n-1}$ with n>1) O2' atom and the P_α atom of the AMP-donor (ATP). After one catalytic step, the newly formed $25A_n$ oligoadenylate rebinds to the acceptor site, where the reaction continues (chain elongation). Initially, an ATP molecule has to bind to the acceptor site ("*R*" in AMP-acceptor represents the diphosphate group). In subsequent chain elongation reaction steps, the acceptor is pppAp(2'-5')A ("*R*" denotes ATP) and later longer oligoadenylates ("*R*" then denotes longer chains).

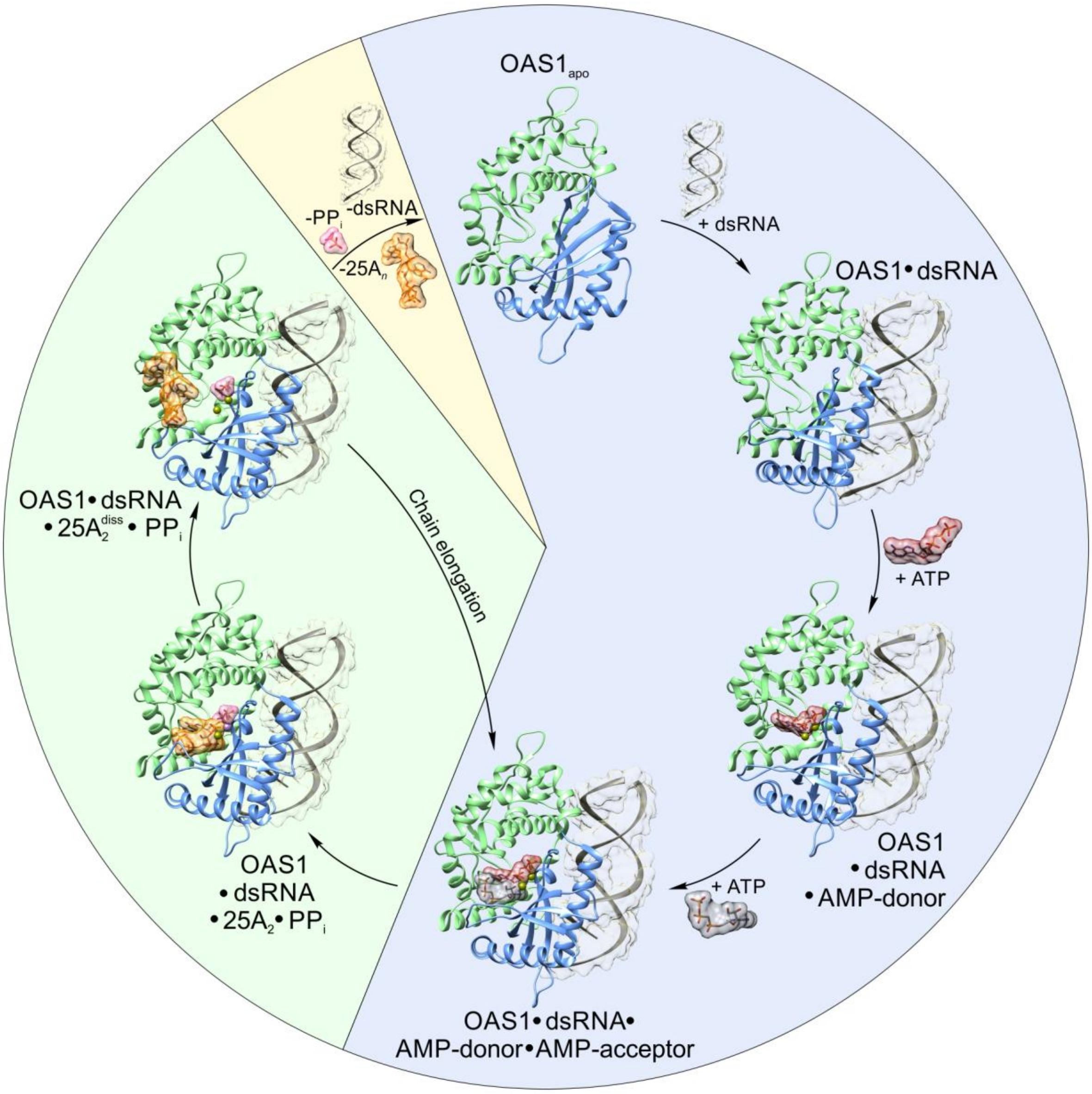

Figure S2: The enzymatic cycle of OAS1. As presented in the study of Lohöfener *et al.* [1] (blue background), the binding of OAS1 in the apo form (PDB: 4RWQ) to dsRNA results in crucial conformational changes activating the enzymatic function of OAS1. The activated OAS1•dsRNA complex (PDB: 4RWP) binds the AMP-donor substrate (PDB: 4RWO). The subsequent binding of the AMP-acceptor completes the formation of the pre-reactive state geometry (PDB: 4RWN) and enables the AMP transfer reaction. In the present study (green background), we determined the post-reactive geometry with both products of OAS1 reaction 25A$_2$ and PP$_i$ bound to the catalytic center (PDB: 9NXS). Our data shows that after the catalytic reaction, the 25A$_2$ main product dissociates first, leaving the PP$_i$ by-product bound at the catalytic center (PDB: 9NY9). The by-product dissociation resets the catalytic center for the subsequent reaction round, where another ATP can bind as an AMP-donor and the synthesized bi-adenylate as a new AMP-acceptor substrate (chain elongation). At the end of its activity cycle, OAS1 dissociates from the dsRNA (orange background).

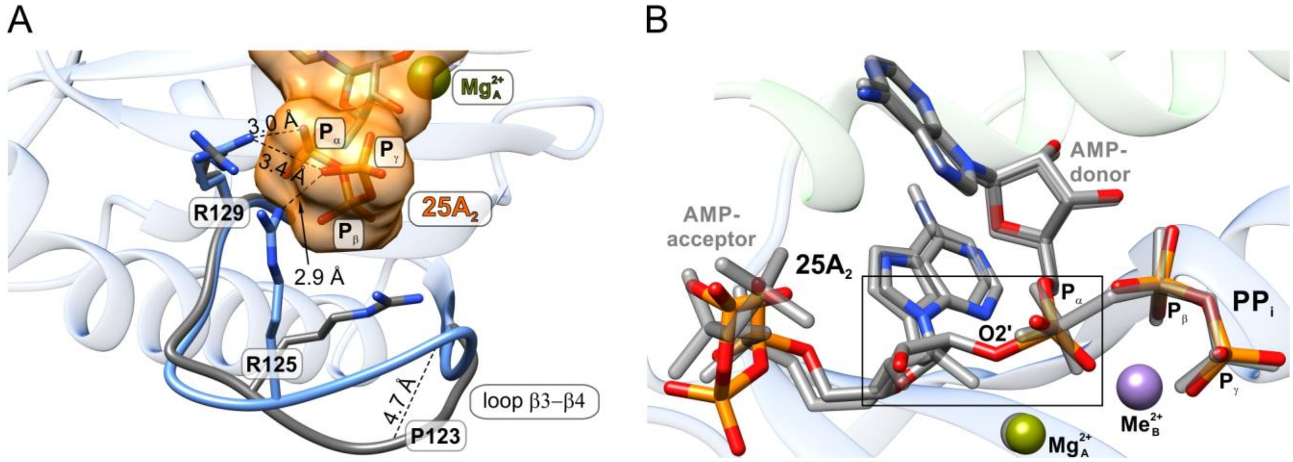

Figure S3: Superposition of key structural elements from the pre-reactive (PDB: 4RWN) and post-reactive pOAS1•dsRNA•25A₂•PPᵢ•Mg^{2+}_A•Mn^{2+}_B complexes. (A) Comparison of the loop β3–β4 from pre-reactive (gray) and post-reactive (blue) complexes. The main structural difference is the 4.7 Å shift in the C_α atom of P123. Additionally, R125 is turned towards $25A_2$ in the post-reactive complex, forming an H-bond with the product's P_γ group. (B) Comparison of the ligands bound to the catalytic center in the pre-reactive (semi-transparent gray) and the post-reactive (colored by element) complexes. The rectangle marks the groups directly involved in the OAS1 reaction, where the new bond between the AMP-acceptor O2' atom and the AMP-donor P_α atom is formed, following the Walden inversion of the donor P_α group.

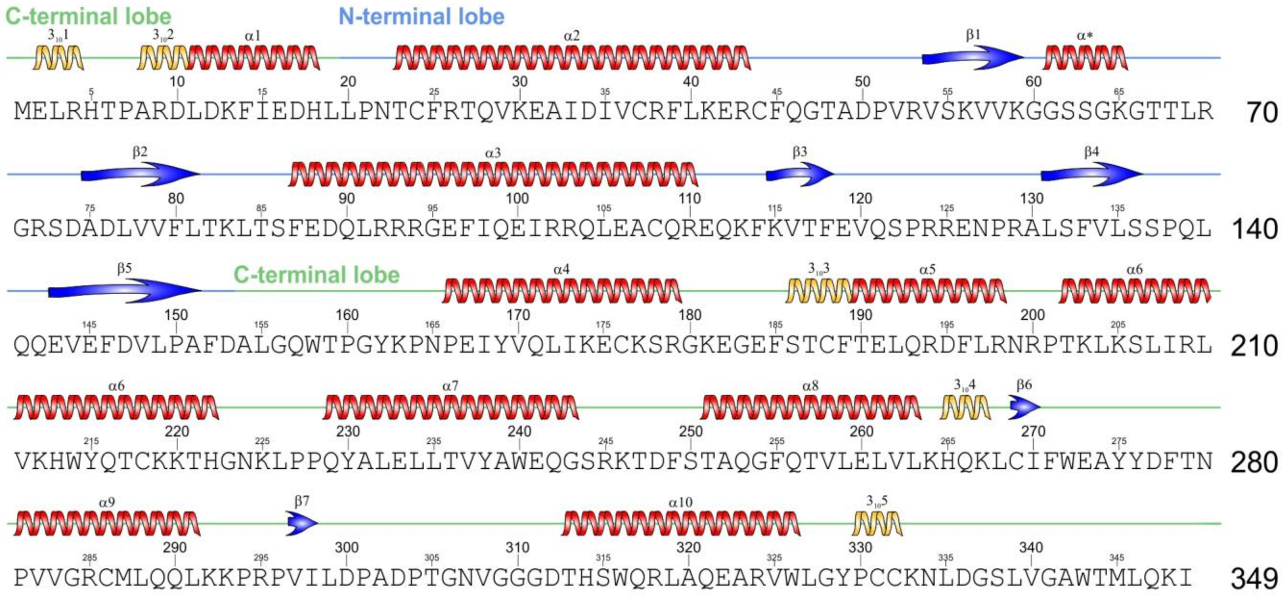

Figure S4: Assignment of secondary structure elements to the amino acid sequence of pOAS1 based on the pre-reactive state complex from Lohöfener *et al.* [1] (PDB: 4RWN).

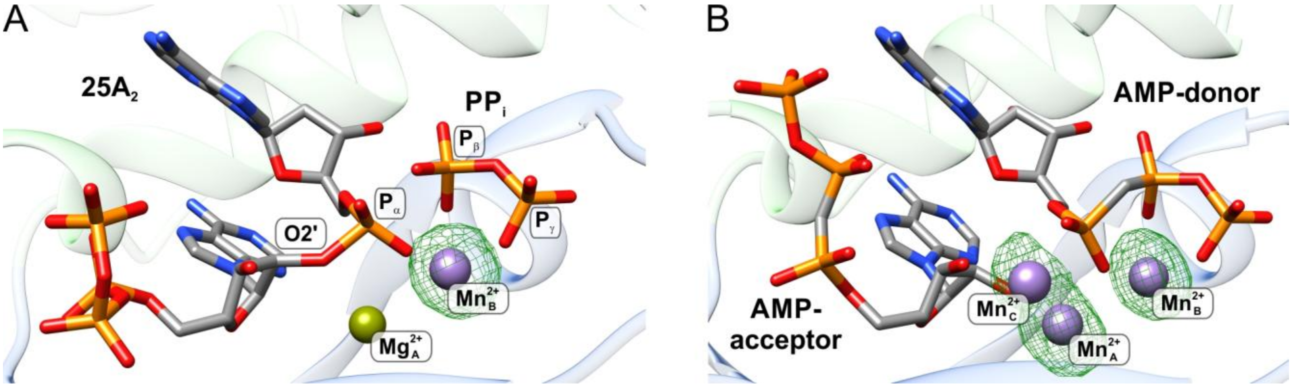

Figure S5: Anomalous density at metal binding sites in complexes pOAS1•dsRNA•$25A_2$•PP_i•Mg^{2+}_A•Mn^{2+}_B (A) and pOAS1•dsRNA•$ApCpp_2$•$Mn^{2+}_{A/B}$•Mn^{2+}_C(B), contoured at 3σ level. The anomalous peaks indicate the binding sites of the manganese ions.

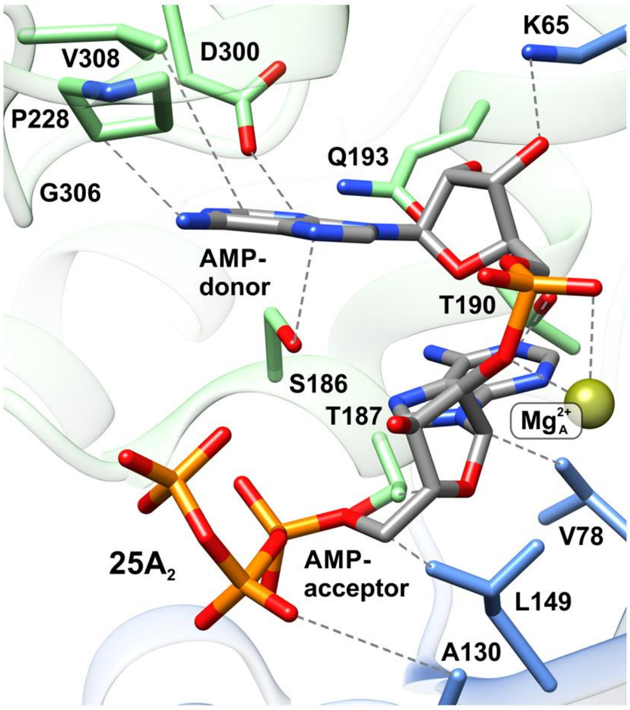

Figure S6: Interactions of AMP donor and AMP acceptor nucleosides in the post-reactive OAS1 complex. The AMP donor nucleoside interacts with K65 (α*), S186 (helix $3_{10}3$), Q193 (α5), P228 (loop α6– α7), D300, and G306–V308 (loop β7– α10). The AMP acceptor nucleoside is coordinated by V78 (β2), A130 (loop β3– β4), L149 (β5), S186–T187 (helix $3_{10}3$), T190, and Q193 (α5), and is additionally stabilized by the $\mathrm{Mg}_{\mathrm{A}}^{2+}$ ion. Hydrogen bonds and metal-coordination interactions are shown as dashed lines.

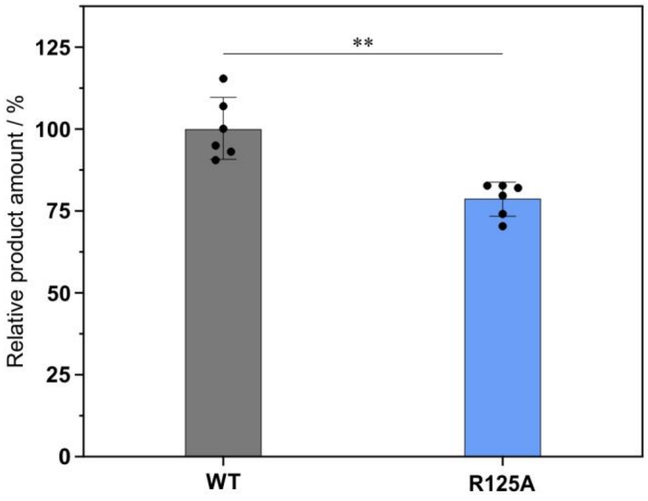

Figure S7: Relative amount of oligoadenylate products synthesized by pOAS1 wild-type (WT) or R125A mutant. Data from six individual experiments were normalized to the mean amount of products generated by the WT protein (100 %) and are shown as mean ± SD. Statistical significance was assessed by unpaired t-test.

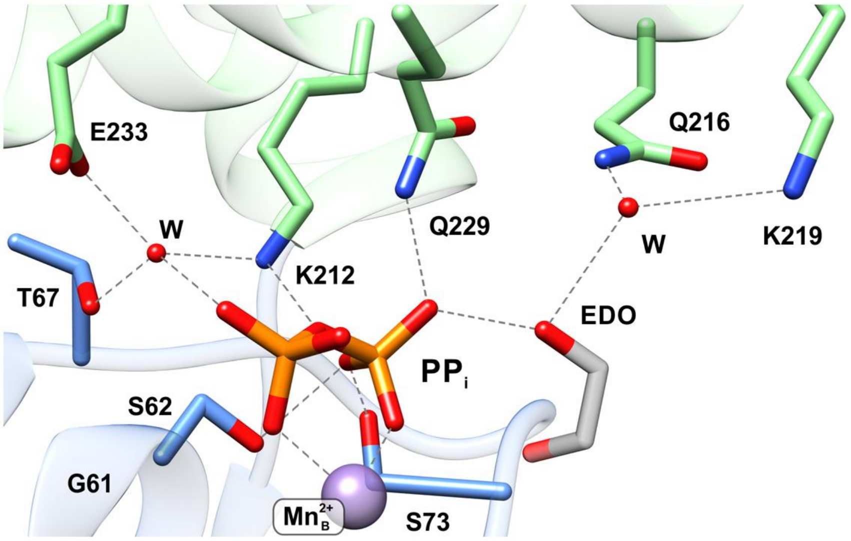

Figure S8: Stabilization of the PPi by-product in the post-reactive OAS1 complex. The by-product forms direct contacts with residues G61–S62 and S73 (α∗), K212 (α6), and Q229 (α7), and is additionally stabilized by coordination to the Mn_B^{2+} ion and interaction with one ethylene glycol molecule. Hydrogen bonds and metal-coordination interactions are depicted as dashed lines.

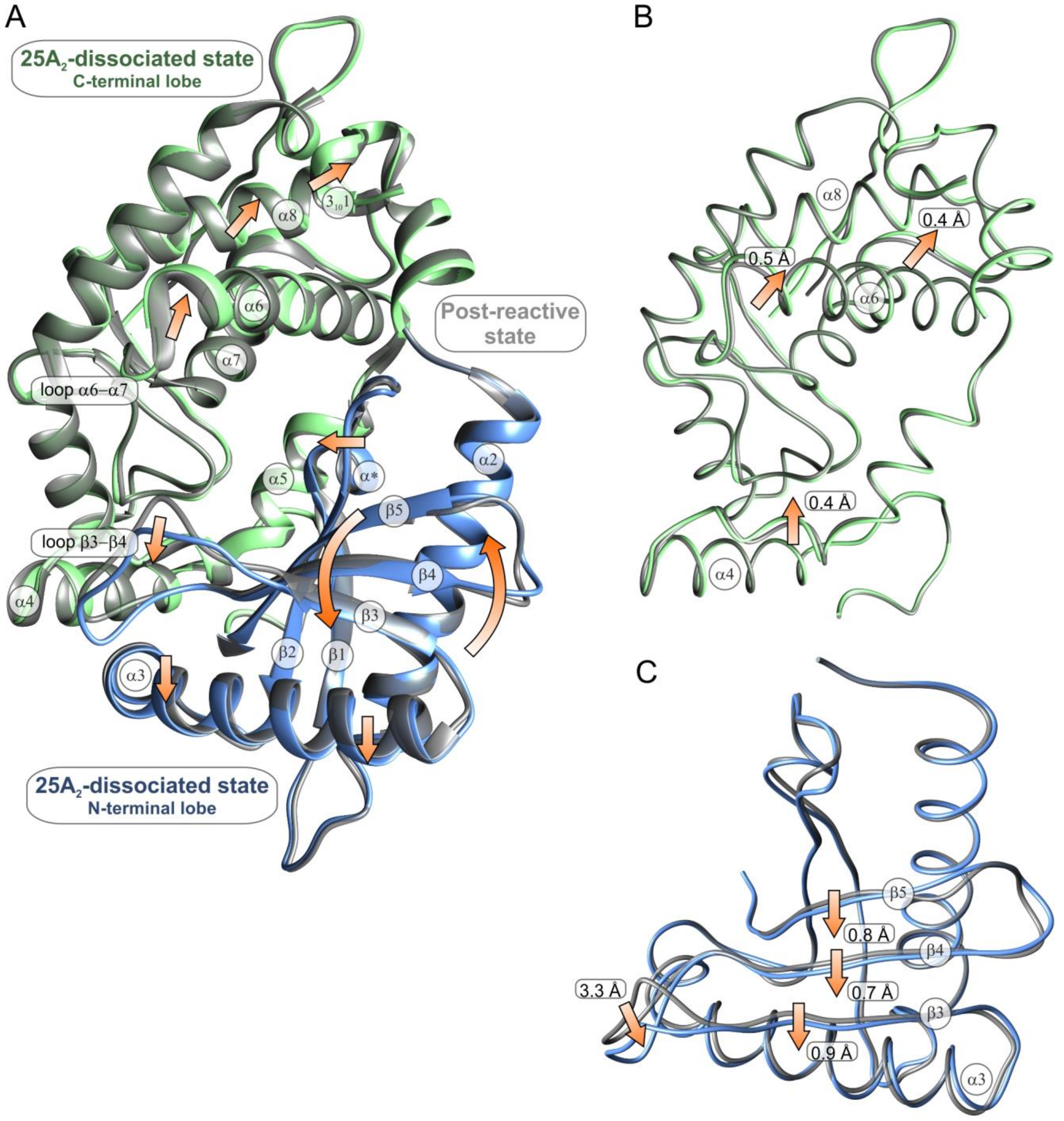

Figure S9: Superposition of the post-reactive pOAS1•dsRNA•25A₂•PPi•Mg_A^{2+}•Mn_B^{2+} and 25A₂-dissociated pOAS1•dsRNA•$25A_2^{diss}$•PPi•$Mg_{A/B}^{2+}$ complexes (A). Orange arrows indicate directions of conformational shifts between the complexes. (B) Close-up view of the C-terminal lobe showing the smaller rearrangements (≤ 0.5 Å for Cα atoms) that collectively contribute to expansion of the active site cavity. (C) Close-up view of the N-terminal lobe highlighting the displacement of the central β-sheet away from the nucleic acid (up to ~1.0 Å for Cα atoms). This shift also involves the catalytic center, while preserving its internal geometry.

A B

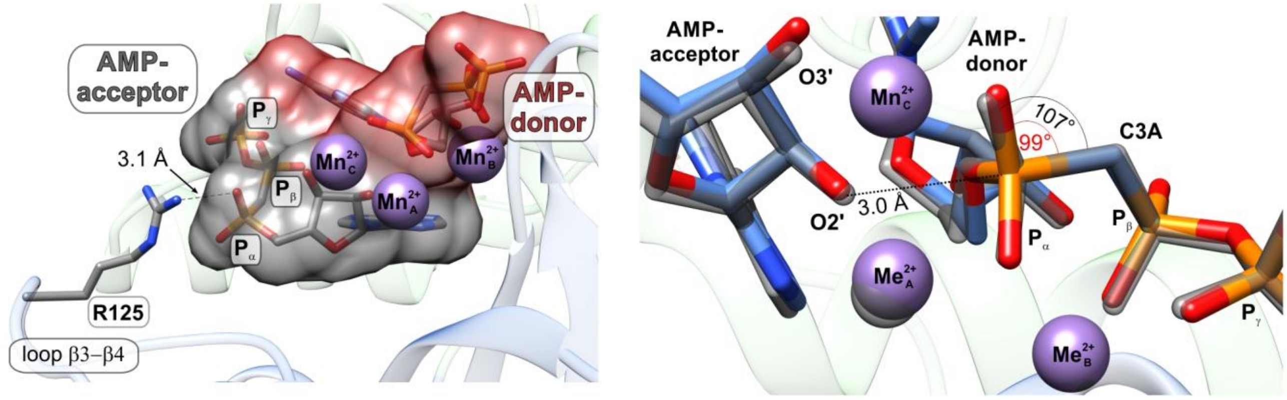

Figure S10: (A) Hydrogen bond between R125 and the AMP-acceptor in the Mn^{2+}-bound pre-reactive state complex of pOAS1 (pOAS1•dsRNA•ApCpp$_2$•$Mn^{2+}_{A/B}$•Mn^{2+}_{C}). (B) Superposition of the catalytic center of the native (gray, semi-transparent, PDB: 4RWN) and Mn^{2+}-bound pre-reactive (colored) state complexes of pOAS1, consisting of two non-reactive ATP analogues ApCpp, and the catalytic metal ions (Me^{2+}). The superposition highlights the more open umbrella conformation of the AMP-donor P_α group in the presence of manganese ions.

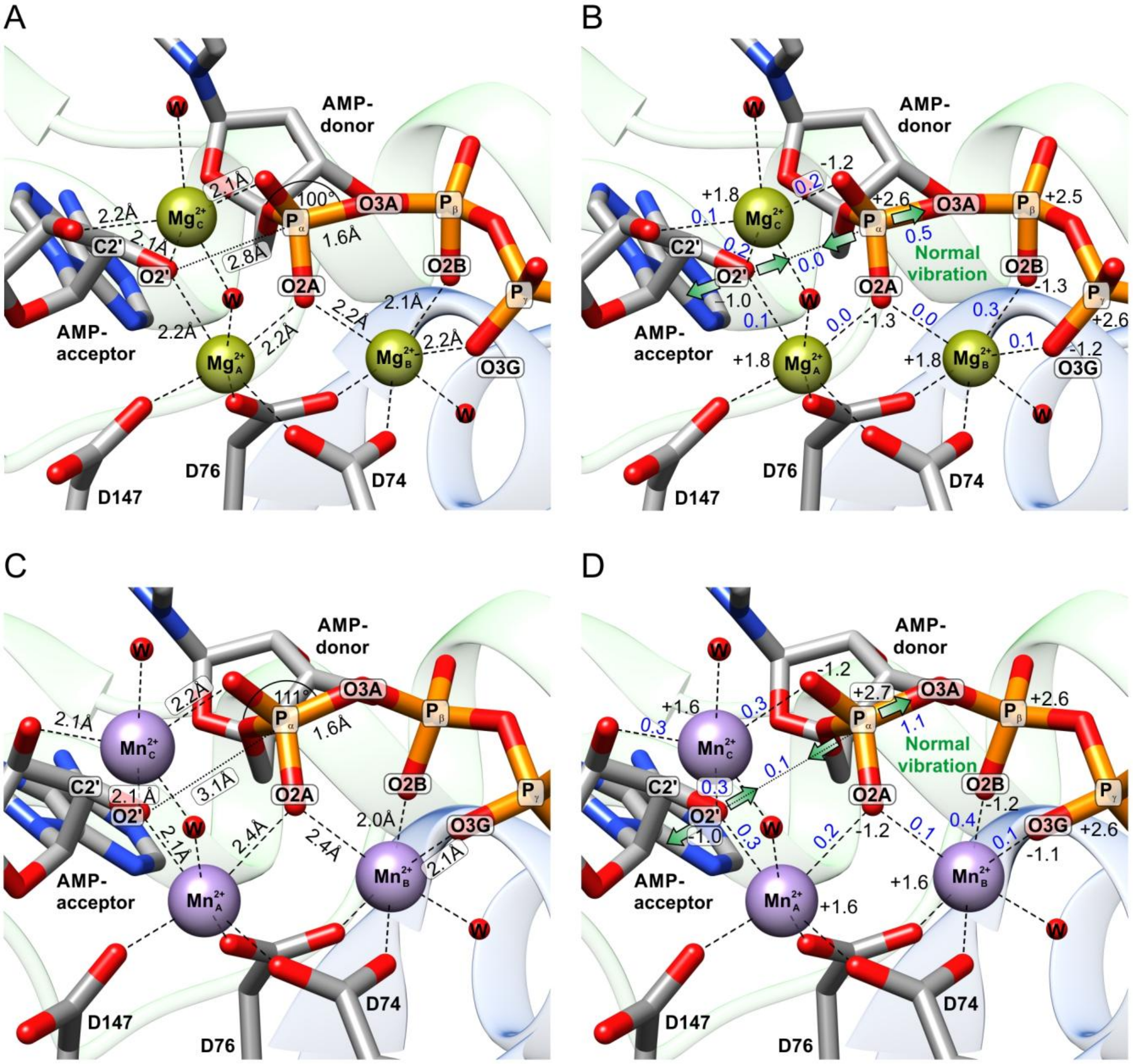

Figure S11: QC-geometry (left column) and electronic properties (right column) of trinuclear Mg^{2+} (A-B) and Mn^{2+} (C-D) catalytic center pOAS1 in the pre-reactive state. The carbon, nitrogen, oxygen, phosphorus, Mg^{2+}, and Mn^{2+} atoms are shown in grey, blue, red, orange, green, and magenta colors, respectively. Hydrogen atoms are omitted for clarity. The DFT NBO charges and bond orders in the right column are shown in black and blue, respectively. The green arrows in panel (B) indicate the QM normal mode, which describes the reaction pathway of OAS.

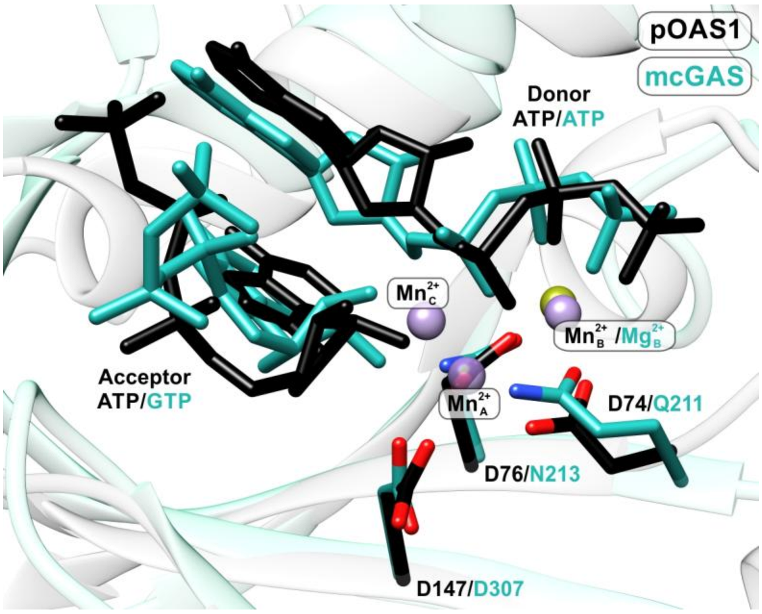

Figure S12: The superposition of the pre-reactive pOAS1•dsRNA•ApCpp$_2$•Mn$^{2+}_{A/B}$•Mn$^{2+}_{C}$complex structure with the pre-reactive state of cGAS (PDB: 7UXW) demonstrates the possibility that cGAS can also form a trinuclear catalytic metal center.

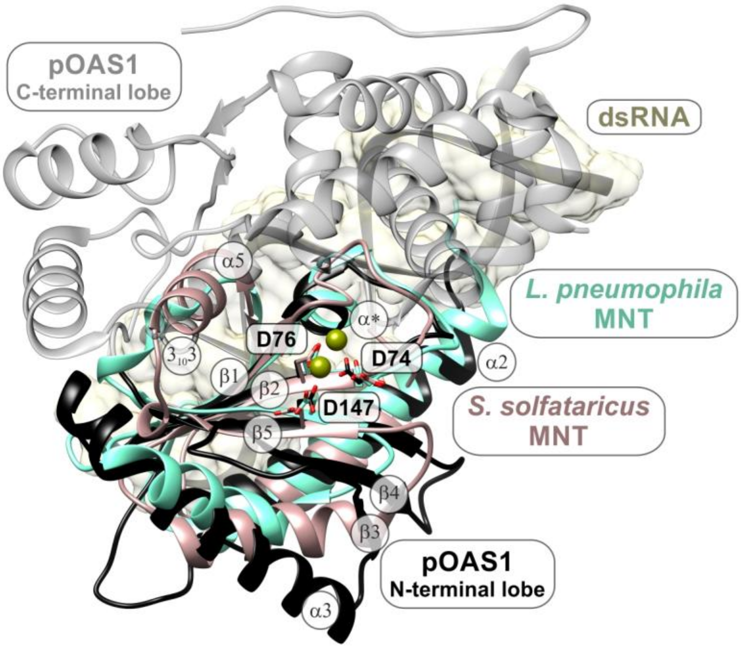

Figure S13: The superposition of pOAS1 pre-reactive state complex (PDB: 4RWN, substrates are removed), a putative (minimal) nucleotidyltransferase from *S. solfataricus* (PDB: 2RFF), and an MNT from *L. pneumophila* (PDB: 8XEO, chain A). For each protein, the three catalytic triad residues are shown in their respective colors. The secondary structure elements and residue numbers are shown for the pOAS1 complex. Mg^{2+} ions from the pOAS1 pre-reactive state complex are shown in green.

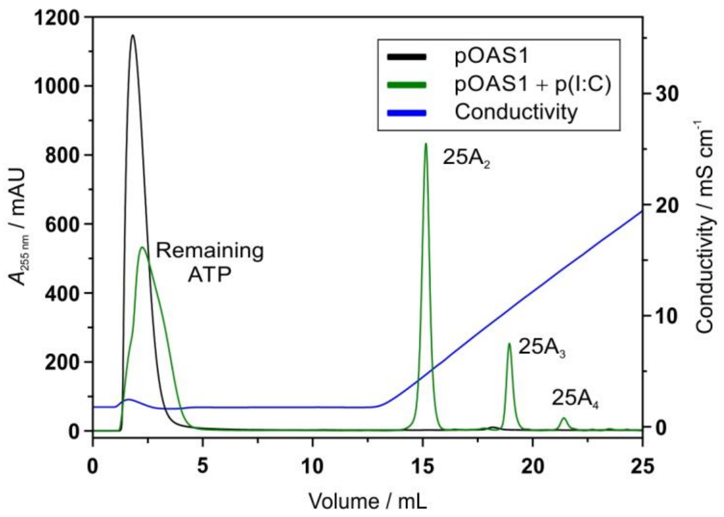

Figure S14: Representative chromatograms from pOAS1 activity test. The curves show the absorbance in the absence (negative control) and presence of 100 ngμL^{-1} p(I:C). The peaks were assigned to the remaining ATP and generated oligoadenylates according to previously published data [10,11].

Supplementary References

(1) Lohöfener, J.; Steinke, N.; Kay-Fedorov, P.; Baruch, P.; Nikulin, A.; Tishchenko, S.; Manstein, D. J.; Fedorov, R. The Activation Mechanism of 2′-5′-Oligoadenylate Synthetase Gives New Insights into OAS/CGAS Triggers of Innate Immunity. *Structure* **2015**, *23* (5), 851–862. https://doi.org/10.1016/j.str.2015.03.012.

(2) Donovan, J.; Dufner, M.; Korennykh, A. Structural Basis for Cytosolic Double-Stranded RNA Surveillance by Human Oligoadenylate Synthetase 1. *Proc. Natl. Acad. Sci. U. S. A.* **2013**, *110* (5), 1652–1657. https://doi.org/10.1073/pnas.1218528110.

(3) Culot, P.; Dive, G.; Nguyen, V. H.; Ghuysen, J. M. A Quasi-Newton Algorithm for First-Order Saddle-Point Location. *Theor. Chim. Acta* **1992**, *82* (3–4), 189–205. https://doi.org/10.1007/BF01113251.

(4) Helgaker, T. Transition-State Optimizations by Trust-Region Image Minimization. *Chem. Phys. Lett.* **1991**, *182* (5), 503–510. https://doi.org/10.1016/0009-2614(91)90115-P.

(5) Baker, J. An Algorithm for the Location of Transition States. *J. Comput. Chem.* **1986**, *7* (4), 385–395. https://doi.org/10.1002/jcc.540070402.

(6) Stein, R. L. Kinetic and Mechanistic Studies of Human Oligoadenylate Synthetase 1. *Biochemistry* **2024**, *63* (20), 2670–2681. https://doi.org/10.1021/acs.biochem.4c00311.

(7) Führing, J.; Cramer, J. T.; Routier, F. H.; Lamerz, A.-C.; Baruch, P.; Gerardy-Schahn, R.; Fedorov, R. Catalytic Mechanism and Allosteric Regulation of UDP-Glucose Pyrophosphorylase from Leishmania Major. *ACS Catal.* **2013**, *3* (12), 2976–2985. https://doi.org/10.1021/cs4007777.

(8) Ivanov, I.; Tainer, J. A.; McCammon, J. A. Unraveling the Three-Metal-Ion Catalytic Mechanism of the DNA Repair Enzyme Endonuclease IV. *Proceedings of the National Academy of Sciences* **2007**, *104* (5), 1465–1470. https://doi.org/10.1073/pnas.0603468104.

(9) Perera, L.; Freudenthal, B. D.; Beard, W. A.; Shock, D. D.; Pedersen, L. G.; Wilson, S. H. Requirement for Transient Metal Ions Revealed through Computational Analysis for DNA Polymerase Going in Reverse. *Proceedings of the National Academy of Sciences* **2015**, *112* (38). https://doi.org/10.1073/pnas.1511207112.

(10) Wang, Y.; Holleufer, A.; Gad, H. H.; Hartmann, R. Length Dependent Activation of OAS Proteins by DsRNA. *Cytokine* **2020**, *126*, 154867. https://doi.org/10.1016/j.cyto.2019.154867.

(11) Eskildsen, S.; Justesen, J.; Schierup, M. H.; Hartmann, R. Characterization of the 2’-5’-Oligoadenylate Synthetase Ubiquitin-like Family. *Nucleic Acids Res.* **2003**, *31* (12), 3166–3173. https://doi.org/10.1093/nar/gkg427.

(12) Froimowitz, M. HyperChem: A Software Package for Computational Chemistry and Molecular Modeling. *Biotechniques* **1993**, *14* (6), 1010–1013.
